# Dopamine transporter and synaptic vesicle sorting defects underlie auxilin-associated Parkinson’s disease

**DOI:** 10.1016/j.celrep.2023.112231

**Published:** 2023-03-14

**Authors:** D.J. Vidyadhara, Mahalakshmi Somayaji, Nigel Wade, Betül Yücel, Helen Zhao, N. Shashaank, Joseph Ribaudo, Jyoti Gupta, TuKiet T. Lam, Dalibor Sames, Lois E. Greene, David L. Sulzer, Sreeganga S. Chandra

**Affiliations:** 1 Department of Neurology, Yale University, New Haven, CT, USA; 2 Department of Neuroscience, Yale University, New Haven, CT, USA; 3 Department of Psychiatry, Columbia University, New York, NY, USA; 4 Division of Molecular Therapeutics, New York State Psychiatric Institute, New York, NY, USA; 5 Department of Computer Science, Columbia University, New York, NY, USA; 6 New York Genome Center, New York, NY, USA; 7 Keck MS and Proteomics Resource, Departments of Molecular Biophysics and Biochemistry, Yale University, New Haven, CT, USA; 8 Department of Chemistry and Neuro Technology Center, Columbia University, New York, NY, USA; 9 Laboratory of Cell Biology, NHLBI, National Institutes of Health, Bethesda, MD, USA; 10 Departments of Neurology and Pharmacology, Columbia University, New York, NY, USA; 11 Aligning Science Across Parkinson’s (ASAP) Collaborative Research Network, Chevy Chase, MD, USA; 12 Program in Cellular Neuroscience, Neurodegeneration and Repair, Yale University, New Haven, CT, USA; 13 Lead contact

## Abstract

Auxilin participates in the uncoating of clathrin-coated vesicles (CCVs), thereby facilitating synaptic vesicle (SV) regeneration at presynaptic sites. Auxilin (*DNAJC6/PARK19*) loss-of-function mutations cause early-onset Parkinson’s disease (PD). Here, we utilized auxilin knockout (KO) mice to elucidate the mechanisms through which auxilin deficiency and clathrin-uncoating deficits lead to PD. Auxilin KO mice display cardinal features of PD, including progressive motor deficits, α-synuclein pathology, nigral dopaminergic loss, and neuroinflammation. Significantly, treatment with L-DOPA ameliorated motor deficits. Unbiased proteomic and neurochemical analyses of auxilin KO brains indicated dopamine dyshomeostasis. We validated these findings by demonstrating slower dopamine reuptake kinetics *in vivo*, an effect associated with dopamine transporter misrouting into axonal membrane deformities in the dorsal striatum. Defective SV protein sorting and elevated synaptic autophagy also contribute to ineffective dopamine sequestration and compartmentalization, ultimately leading to neurodegeneration. This study provides insights into how presynaptic endocytosis deficits lead to dopaminergic vulnerability and pathogenesis of PD.

## In brief

Auxilin participates in clathrin uncoating to facilitate presynaptic endocytosis. Loss-of-function mutations of auxilin (*PARK19*) cause Parkinson’s disease. Using auxilin KO mice, Vidyadhara et al. show that synaptic vesicle sorting deficits, cytoplasmic dopamine accumulation, dopamine transporter mistrafficking, and synaptic autophagic overload may lead to pathogenesis of Parkinson’s disease in *PARK19* patients.

## Graphical Abstract

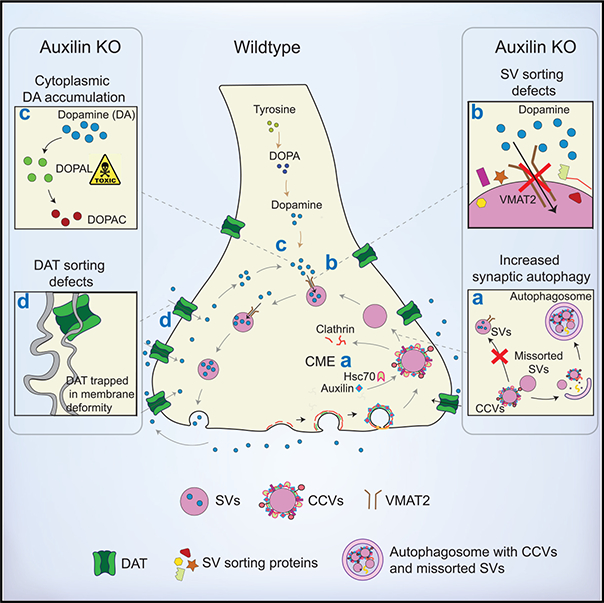

## INTRODUCTION

Presynaptic boutons of dopaminergic (DA) nigrostriatal neurons are sites of initiation for neurodegeneration in Parkinson’s disease (PD).^1^ Nigrostriatal DA neurons have long, hyperbranched axons as well as tonic firing properties that make them reliant on efficient synaptic vesicle (SV) recycling to maintain a steady-state SV pool for neurotransmission.^2^ In presynaptic sites, SV recycling is supported by several endocytic pathways, including clathrin-mediated endocytosis (CME), ultrafast endocytosis, and bulk endocytosis.^3^ Clathrin-coated vesicles (CCVs) are a common intermediate of these different endocytic pathways. CCVs are uncoated by the coordinated action of auxilin (*DNAJC6*) or its ubiquitous homolog cyclin-G-associated kinase (*GAK*), along with synaptojanin-1 (*SNJ1*), endophilin-A1 (*ENDOA1*), and the chaperone Hsc70.^2^ Interestingly, mutations in all four genes (*DNAJC6, GAK, SNJ1, ENDOA1*) have been identified as causal or risk alleles for PD/parkinsonism,^4–8^ suggesting a major role for altered clathrin uncoating in the initiation of DA presynaptic degeneration and pathogenesis of both familial and sporadic PD.^2^ Animal models carrying these mutations also show clathrin uncoating and presynaptic endocytosis defects;^9–11^ however, how these disturbances result in characteristics of PD is not fully clear.

Auxilin is a brain-specific heat-shock protein 40family co-chaperone that functions to uncoat CCVs to nascent SVs by recruiting Hsc70.^12,13^ Unlike other co-chaperones, auxilin has a limited number of substrates^14^ and only one described function, i.e., clathrin uncoating. Loss-of-function, autosomal recessive mutations of the auxilin gene (*PARK19*) cause juvenile early-onset PD.^4,15–20^ A recent study shows that *PARK19* mutations also occur in late-onset PD patients.^21^ *In vivo* presynaptic dopamine transporter (DAT) imaging of a *PARK19* patient revealed DA terminal loss, which supports a parkinsonian diagnosis and suggests that clathrin-uncoating deficits impact DA presynaptic sites. ^15^ Furthermore, LRRK2 mutations, a common genetic cause of PD, may exert some of its pathological actions through auxilin. In LRRK2 patient induced pluripotent stem cell derived DA neurons, LRRK2 phosphorylation of auxilin led to decreased auxilin levels and clathrin binding, resulting in accumulation of oxidized dopamine and α-synuclein overexpression.^22^ Whether loss-of-function mutations in auxilin can also trigger PD through these mechanisms is unknown. Nonetheless, these new-found links between auxilin and LRRK2 implicate a role for auxilin in both familial and sporadic PD. The relevance of auxilin to all forms of PD is underscored by the finding that GAK (*DNAJC26*) is a risk allele for sporadic PD.^5^

Prior to the discovery of auxilin *PARK19* mutations, auxilin knockout (KO) mice were generated and characterized for CME deficits.^23^ Analysis of synapses in deep cerebellar nuclei of adult auxilin KO mice revealed accumulation of CCVs and empty clathrin cages (lacking SV membrane). Similar ultrastructural alterations were seen *in vitro* in primary neurons and were accompanied by defective SV endocytosis.^23^ These findings confirmed that auxilin functions in clathrin uncoating. Although *PARK19* patients have loss-of-function mutations and not a deletion of *DNAJC6/auxilin*, auxilin KO mice can provide crucial insights into how a primary deficit in clathrin uncoating leads to selective vulnerability of DA neurons and PD. Thus, we characterized auxilin KO mice for age-dependent nigrostriatal degenerative changes and investigated the underlying mechanisms. Our results demonstrate that in auxilin KO mice, cytoplasmic dopamine accumulation, DAT mistrafficking, SV sorting deficits, and autophagic overload in dorsal striatal DA presynaptic sites initiate behavioral and histochemical signatures of PD.

## RESULTS

### Auxilin KO mice develop age-dependent PD-like behavioral abnormalities

**Figure 1.**
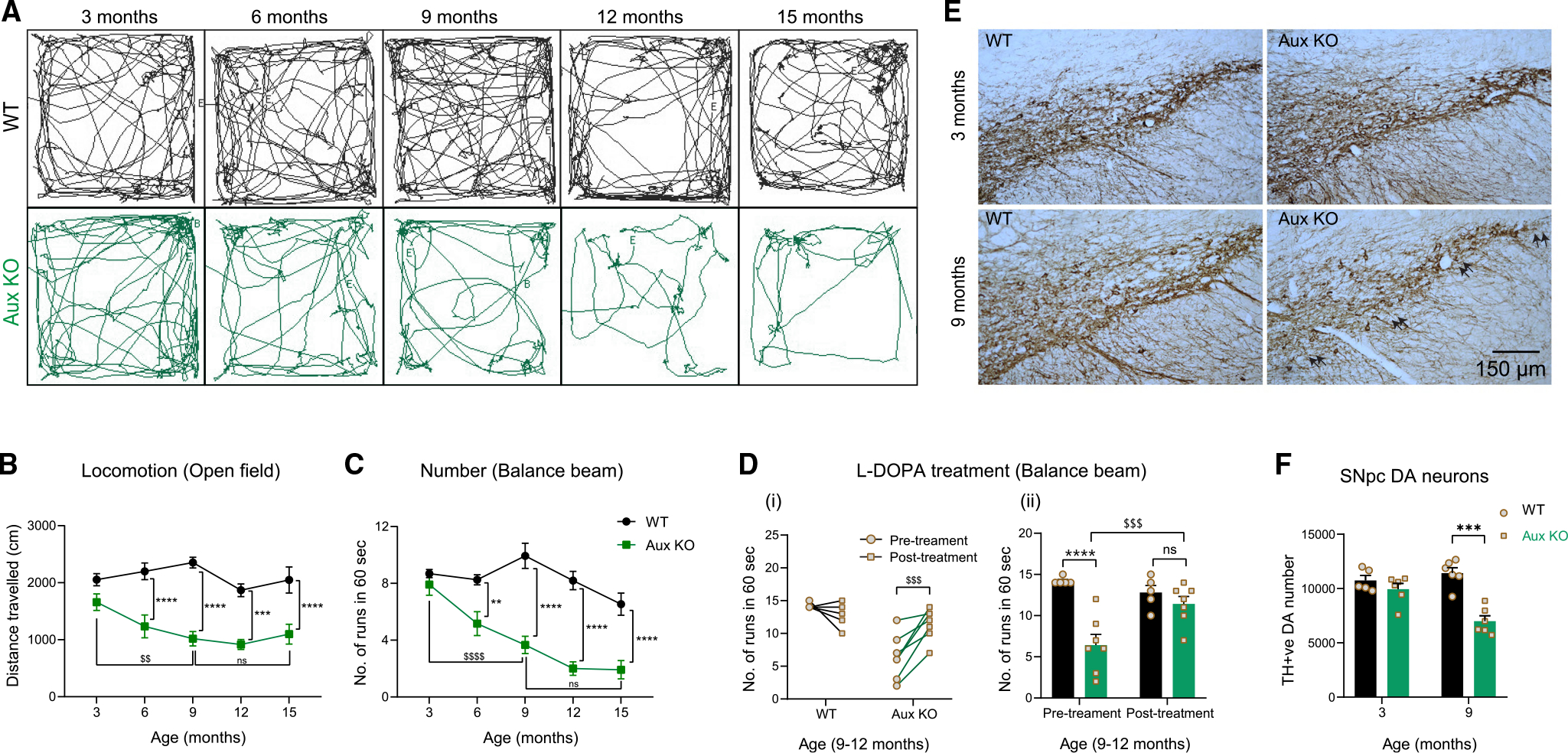
Auxilin KO mice develop progressive motor behavior deficits that are accompanied by nigral DA neuronal loss (A) Longitudinal open field locomotor behavior tracings of WT and auxilin KO (Aux KO) mice. (B) Distance traveled in open field as a function of age showing a progressive diminishment in locomotion in Aux KO mice (n=12−16/genotype). (C) Number of runs performed in 1 min on a balance beam. Performance of Aux KO mice decreased with age, with a significant difference after 9 months. (D) (i) Balance beam performance of individual WT and Aux KO mice at 9–12 months of age before and after treatment with L-DOPA. (ii) Note a significant recovery in Aux KO mice in balance beam performance comparable with that of WT mice post treatment. (E) Representative images showing TH^+^ SNpc DA neurons in WT and Aux KO midbrain sections at 3 and 9 months of age. Fewer DA neurons (arrows) were present in the SNpc of Aux KO mice at 9 months. Scale bar, 150 μm. (F) Unbiased stereological counting of SNpc DA neurons. A significant (~40%) loss of DA neurons is seen in 9-month-old Aux KO mice (n=5−6/genotype). Data are presented as mean ± SEM. ns, not significant; **p < 0.01, ***p < 0.001, ****p < 0.0001, ^$$^p < 0.01, ^$$$^p < 0.001, ^$$$$^p < 0.0001. (**comparison between genotypes, ^$^comparison between ages/time points)

We performed a battery of behavioral assays to evaluate whether auxilin KO mice develop age-dependent motor behavior abnormalities akin to PD patients. We monitored cohorts of wild-type (WT, C57BL/6J) and auxilin KO (congenic B6.-*Dnajc6*^*tm1Legr*^) mice longitudinally, assessing behavior from 3 to 15 months of age. Locomotion and ambulatory behaviors were evaluated by the open field test. Auxilin KO mice behave like WT mice at 3 months of age but show a significant age-dependent decrease in overall distance traveled, starting at 9 months (Figures 1A and 1B). Next, we tested the same cohorts on a balance beam to evaluate motor coordination. We assessed the ability of mice to traverse a raised narrow beam by measuring the number of runs performed in 1 min (Figure 1C) and the time taken to cross the beam (Figure S1A). The performance of auxilin KO mice was comparable to WT controls at 3 months but deteriorated at later ages with a significant deficit emerging at 9 months (Figures 1C and S1A; Video S1). These results suggest that auxilin KO mice are normal at 3 months but become symptomatic by 9 months, exhibiting a progressive decrement in motor function at later ages. Remarkably, symptomatic 9- to 12-month-old auxilin KO mice when treated with L-DOPA showed complete recovery of their performance on the balance beam apparatus (Figures 1Di,ii and S1bi,ii). We could only see a trend toward recovery in locomotion in the open field in auxilin KO mice after L-DOPA treatment (Figure S1C), probably because the results were confounded by the short time interval between tests, which hampers novelty-induced exploration.

To evaluate the contribution of brain areas other than the nigrostriatal pathway in auxilin KO mice, we performed Rotarod, hindlimb clasping, and limb grip strength behavior assays. The performance of auxilin KO mice on the Rotarod and grip strength meter was comparable with that of WT mice (Figures S1G, S1I, and S1J). Auxilin KO mice at 6 and 9 months of age showed some hindlimb clasping (Video S2); however, the time spent clasping was not significantly different from that taken by WT mice (Figure S1H). Auxilin KO mice did not exhibit anxiety-like behavior as evaluated by fecal pellet expulsion (Figure S1D) and time spent in the inner and outer circle of an open field apparatus (Figures S1E and S1F). Additionally, there was no difference in body weight between genotypes, suggesting normal food intake (Figure S1K). Together, these observations indicate that auxilin KO mice develop age-dependent, selective motor deficits, consistent with *PARK19* and PD patients.

### Aged auxilin KO mice faithfully replicate cardinal histopathological signatures of PD

Motor symptoms in PD manifest because of the degeneration of DA neurons in the substantia nigra pars compacta (SNpc) when DA loss reaches a threshold of 40%–50%.^24^ We performed stereological quantitation of SNpc DA neurons, which are immunoreactive to tyrosine hydroxylase (TH), in WT and auxilin KO mice to understand the cellular basis for the motor symptoms we observed. No change in DA neuron numbers was seen in 3-month-old auxilin KO mice (Figures 1E and 1F). However, at the symptomatic age of 9 months, a significant loss of DA neurons was observed (~40%) (Figures 1E, 1F, and S2A), which was also reflected in neuronal counts after immunostaining for neuronal nuclei (NeuN) (Figures S2B and S2C). A similar level of DA neuronal loss was seen in 15-month-old auxilin KO mice (Figures S2D and S2E). Neuronal loss was distributed throughout the SNpc (Figure 1E, arrows), as observed in models of α-synuclein overexpression^25^ and vesicular dopamine storage deficits,^26^ but dissimilar to the ventrolateral loss seen in neurotoxic models.^27^

**Figure 2.**
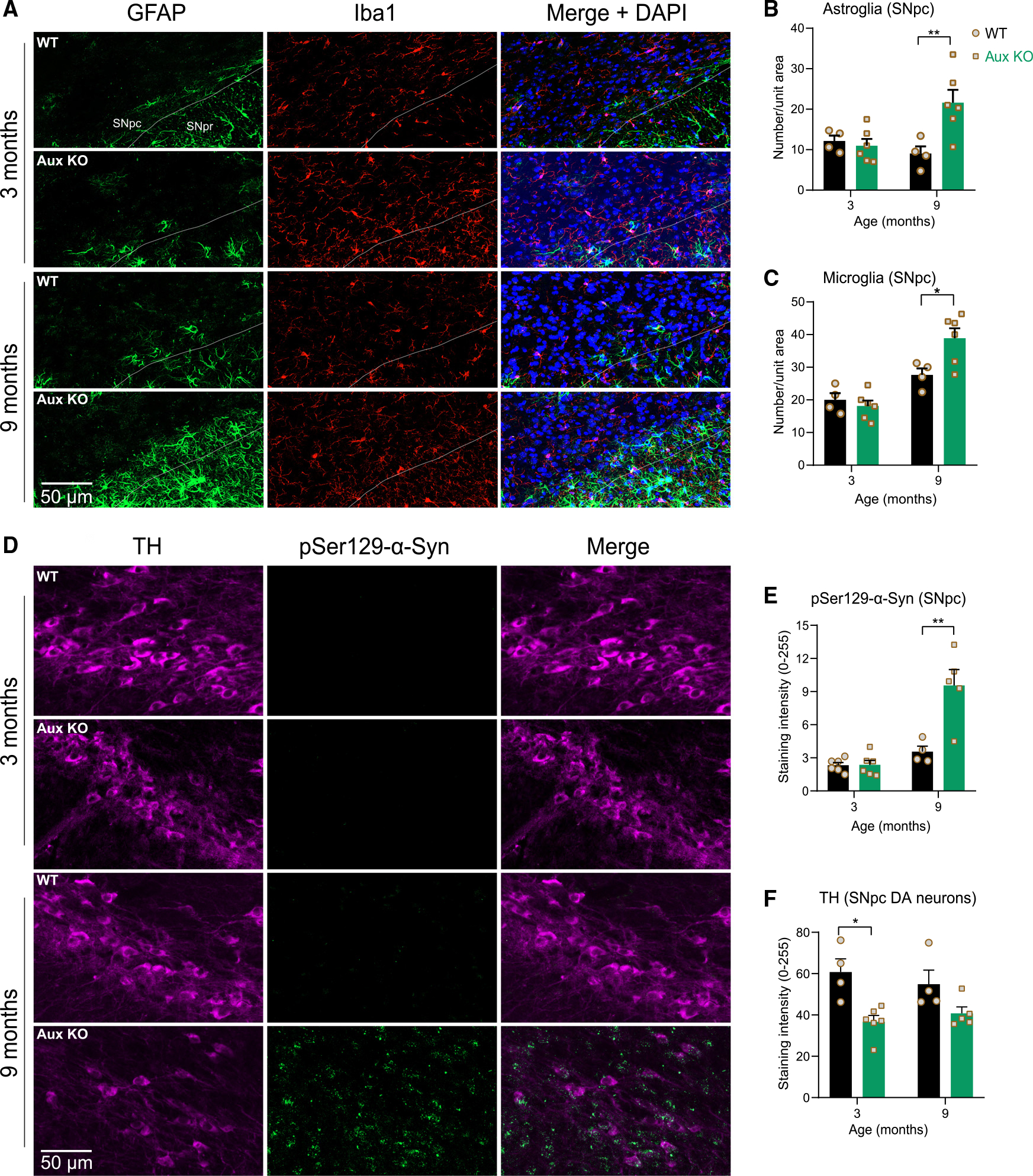
Aged auxilin KO mice exhibit gliosis and α-synuclein pathology (A) Representative images of SNpc and SN pars reticulata (SNpr) of WT and Aux KO mice at 3 and 9 months of age (n=5−6/genotype) immunostained for the astroglial marker GFAP (green) and microglial marker Iba1 (red). Dashed line demarcates SNpc from SNpr. Scale bar, 50 μm. (B) Quantitation of SNpc GFAP-positive cells shows a significant astrogliosis at 9 months. (C) Quantitation of Iba1^+^ cells shows microgliosis in the SNpc of Aux KO mice at 9 months. (D) Representative images of the SNpc immunostained for pSer129-α-synuclein (green), co-stained with DA marker TH (magenta). Scale bar, 50 μm. (E) Quantitation of pSer129-α-synuclein-positive punctate aggregates in SNpc, which showed an increase in 9-month-old Aux KO mice, but not at 3 months of age. (F) Quantitation of TH staining intensity per DA neuron of SNpc, which showed a moderate decrease in Aux KO mice, suggesting retention of TH phenotype in the surviving neurons. Data are presented as mean ± SEM. *p < 0.05, **p < 0.01.

To assess whether neurodegeneration was accompanied by neuroinflammation, we immunostained for glial fibrillary acidic protein (GFAP), an astroglial marker, and ionized calcium-binding adapter molecule 1 (Iba1), a microglial marker (Figure 2A). Significant astrogliosis and microgliosis was seen in auxilin KO mice at 9 months but not at 3 months (Figures 2A–2C). Next, we tested whether auxilin KO brains exhibit α-synuclein pathology, a hallmark of PD.^24^ We utilized a pSer129-α-synuclein antibody that is specific for detection of phosphorylated α-synuclein associated with disease pathology.^28^ Strikingly, auxilin KO brains showed age-dependent α-synuclein pathology. pSer 129-α-synuclein immunostaining was seen in the TH-positive (TH^+^) SNpc at 9 months but not at 3 months of age in auxilin KO mice (Figures 2D and 2E). Immunostaining also revealed a moderate decrease in TH expression at 3 months in nigral DA neurons, with no significant change at 9 months of age in auxilin KO mice (Figures 2D and 2F).

To test whether pathology is seen in other brain areas, we examined the ventral tegmental area (VTA) and hippocampus, regions spared in PD. DA neuronal loss in the VTA of auxilin KO was minimal as quantitated by both stereology and FIJI-based counting (Figures S3A–S3D). TH expression was also unaltered (Figures S3C and S3E). pSer129-α-synuclein pathology was seen in the VTA (Figures S3C and S3F) and in the pyramidal cell layer of hippocampus (Figures S4A–S4C) at 9 months of age, but its expression did not reach significance. We monitored neuronal loss in the hippocampus by measuring the thickness of the CA1 pyramidal cell layer, which was preserved in both 3- and 9-month-old auxilin KO mice (Figures S4D and S4E). Additionally, both microglial and astroglial numbers in the hippocampus of auxilin KO mice were also comparable with those in WT mice (Figures S4F–S4I). Taken together, auxilin KO mice develop age-dependent pSer129-α-synuclein pathology, gliosis, and neurodegeneration in a stereotypical pattern, replicating the regional vulnerability of classic PD. Auxilin KO mice are thus a reliable and robust model for PD.

### Proteomic analysis of auxilin KO mouse brains implicate defective dopamine degradation

**Figure 3.**
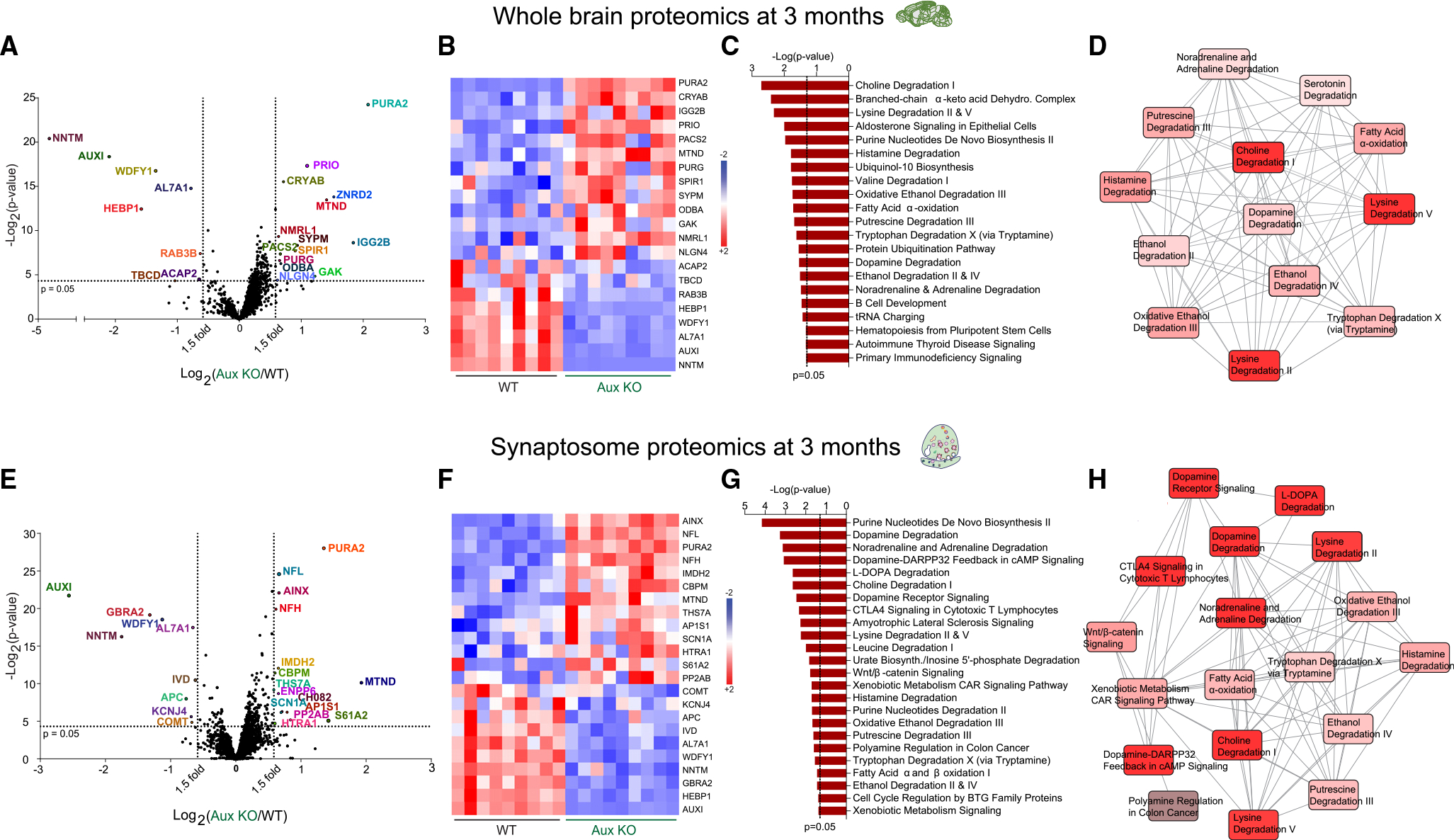
Whole-brain and synaptosome proteomics reveal dopamine degradation dysfunction in young auxilin KO mice (A) Volcano plot of whole-brain proteome of Aux KO compared with WT mice (age 3 months, n=3mice/genotype). Proteins that exhibit a 1.5-fold change (vertical dotted lines) and a p value of 0.05 (Student’s t test) or less (horizontal dotted line) were considered as significantly changed. (B) Heatmap of significantly changed proteins in whole-brain homogenates of WT and Aux KO mice for all nine technical replicates (3 technical replicates/mouse).Red indicates an increased level (+2) and blue indicates a decreased level (−2). (C) Pathways that are significantly (p<0.05) affected in whole brain of Aux KO mice as determined by IPA. (D) Diagram showing the overlap of significantly affected pathways, where intense red depicts most affected and light red depicts moderately affected pathways. (E) Volcano plot of synaptosome proteome of Aux KO compared with WT mice (age 3 months, n=3mice/genotype). (F) Heatmap of significantly changed proteins in synaptosomes from Aux KO mice in comparison with WT mice for each technical replicate. Red indicates an increased level (+2) and blue indicates a decreased level (−2). (G) Significantly affected pathways due to synaptosome proteomic changes as determined by IPA. (H) Overlap of significantly affected pathways showing highly affected (intense red) and moderately affected (light red) pathways.

To gain unbiased insights into the consequences of auxilin loss of function, we performed proteomic analysis on whole-brain and neuronal synaptosome fractions from 3-month-old WT and auxilin KO mice (n = 3/genotype) using label-free quantification mass spectrometry (LFQ-MS). We confirmed the complete absence of auxilin in auxilin KO mouse brains by western blotting of a subset of samples used for proteomics (Figures S5A–S5C). By LFQ-MS we detected 2,851 proteins in the whole-brain proteome, 22 of which were significantly changed in KO samples (Figures 3A and 3B; Table S1). We observed, as expected, decreased auxilin levels and compensatory increases in the auxilin homolog GAK.^23^ Many of the prominent proteins whose levels are changed are linked to PD and neurodegeneration, including RAB3B, TBCD, ACAP2, HEBP1, WDFY1, and NNTM, which are decreased, while CRYAB, PRIO, and NMRL1 are increased in auxilin KO brains (Figures 3A and 3B; Table S1). Ingenuity Pathway Analysis (IPA) revealed that the top pathways were highly overlapping and involve the degradation of lysine, choline, and monoaminergic neurotransmitters, including dopamine (Figures 3C and 3D). Interestingly, a decrease in AL7A1 appears to drive the top canonical pathways (Figure 3C and Table S5). AL7A1 or aldehyde dehydrogenase 7A1 (ALDH7A1) is a multifunctional enzyme which plays a crucial role in detoxification of reactive aldehydes and oxygen species that are generated during monoaminergic neurotransmitter metabolism.^29^ Aldehydes that accumulate because of ALDH7A1 loss of function hinder dopamine synthesis.^30^

Proteomic analysis of whole-brain neuronal synaptosome fractions identified 3,124 proteins, 24 of which were significantly dysregulated (Figures 3E and 3F; Table S2). Along with the expected downregulation of auxilin, auxilin KO mice showed decreased AL7A1, NNTM, and WDFY1, and an upregulation in PURA2 and MTND, proteins that were also significantly changed in whole-brain proteomic data (Figures 3A and 3B). Three neurofilaments, NFL, NFH, and AINX, were upregulated and are candidate biomarkers for axonal damage in PD.^31^ A crucial dopamine metabolizing enzyme, catechol-*o*-methyltransferase (COMT)^32^ was also significantly decreased in synaptosome preparations of auxilin KO mice. HTRA1, PP2A, KCNJ4, and APC are a few of the PD-linked proteins that were also dysregulated (Figures 3E and 3F; Table S2). In all, we found alterations in proteins linked to PD (13 out of 23), including key dopamine metabolism enzymes. This is also evident from the IPA analysis, where the top affected pathways are related to dopamine degradation (Figures 3G and 3H). A high fraction of the canonical pathways predicted for the whole-brain analysis were replicated in IPA analysis for the synaptosome samples (10 out of 21, compare Figures 3G and 3H with 3C and 3D; Table S6), suggesting a major impact on the function of DA synapses upon loss of auxilin.

To evaluate whether the proteomic prediction of disruption in dopamine degradation in young auxilin KO brains (Figure 3) leads to activation of downstream neurodegenerative pathways at an older age, we performed LFQ-MS on synaptosomes from 9-month-old WT and auxilin KO mice. In these preparations, we still observed a compensatory increase in GAK in auxilin KO brains. NFL, AINX, and CADH2 were also upregulated as in the 3-month dataset, reinforcing their potential as biofluid biomarkers of PD^31^ (Figures S6A and S6B). mTOR, a key cell survival and autophagy regulator which helps maintain striatal DA projections^33^ and is linked to PD,^34^ was decreased in auxilin KO mice. RGS6, a critical regulator of dopamine feedback signaling in nigrostriatal DA neurons and a modulator of PD pathology,^35^ was also downregulated (Figures S6A and S6B; Table S4). IPA revealed highly overlapping autophagic pathways such as ILK, P13K/AKT, mTOR, and AMPK signaling, along with oxidative stress, DA signaling, and ubiquitination pathways as dysregulated in auxilin KO synaptosomes at 9 months of age (Figure S6C), which have been directly linked to neurodegeneration in PD.^24^ Together, our proteomic analyses suggest that dysfunction of presynaptic dopamine homeostasis is likely to be an early pathogenic event in auxilin-linked PD.

### Disrupted striatal dopamine homeostasis in auxilin KO mice

**Figure 4.**
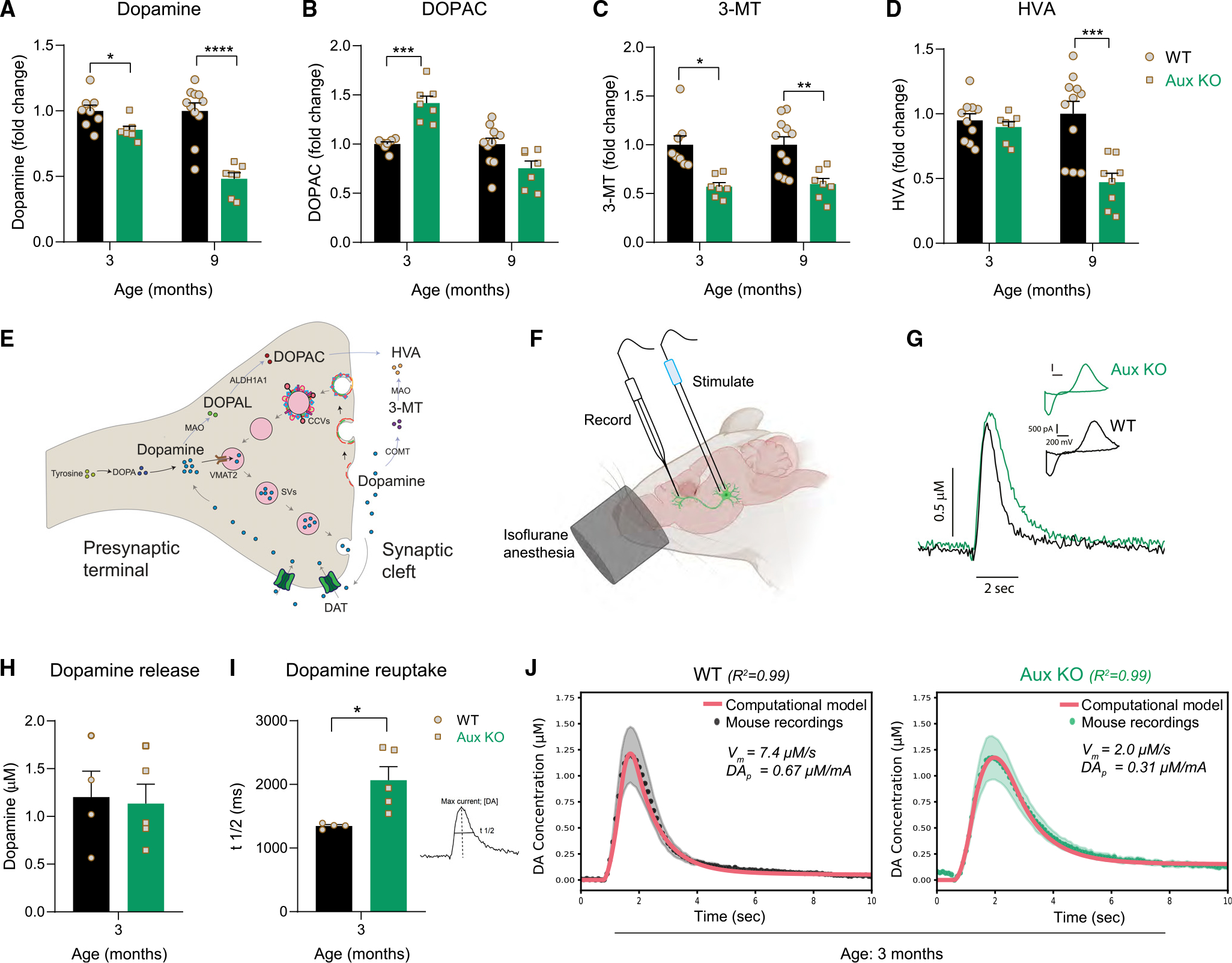
Dopamine catabolism and dopamine reuptake deficits in young auxilin KO mice (A) Dopamine levels in the dorsal striatum of WT and Aux KO mice (n=7−11mice/genotype). (B) DOPAC levels in the dorsal striatum. (C) 3-MT levels in the dorsal striatum. (D) HVA levels in the dorsal striatum. (E) Schematic showing compartmentalization of dopamine and its catabolites in intra- and extra-synaptic space. (F) Schematic showing the location of FSCV recording electrode in the dorsal striatum and the bipolar stimulating electrode in the ventral midbrain of mice under isoflurane anesthesia. (G) Example trace of evoked dopamine release following stimulation of midbrain DA neurons by 30 pulses at a constant 50-Hz frequency in 3-month-old Aux KO and WT mice (scale: y axis, 0.5 μM dopamine; x axis, 2 s). (H) Dopamine release in the dorsal striatum (n=4−5/genotype). (I) Dopamine reuptake in the dorsal striatum. Reuptake kinetics measured by time taken to clear half the dopamine from its peak levels (t_1/2_) was significantly delayed in Aux KO mice. (J) Best fits of computational model of dopamine (DA) release (red lines) to averaged FSCV recordings in the dorsal striatum of WT (black dots; $R^{2}=0.99$, n=4) and Aux KO (green dots; $R^{2}=0.99$, n=5) mice. Black/green ribbons report SEM. Data are presented as mean ± SEM. *p < 0.05, ***p < 0.001, ****p < 0.0001.

To validate the mass spectrometric predictions of altered presynaptic dopamine homeostasis, we measured the levels of dopamine and its metabolites in dorsal striatum of WT and auxilin KO mice using high-performance liquid chromatography (HPLC). Dopamine levels were moderately decreased (14.5%) at 3 months in auxilin KO compared with WT mice, whereas the decrease was more pronounced at 9 months (52%, Figure 4A) when motor deficits are seen (Figure 1). The latter can be attributed to loss of SNpc DA neurons seen at 9 months of age in auxilin KO mice (Figures 1E and 1F) as these neurons project to dorsal striatum. We also measured serotonin levels, which were unchanged in auxilin KO mice (Figure S7A). Next, we evaluated the levels of dopamine metabolites 3,4-dihydroxyphenylacetic acid (DOPAC) and homovanillic acid (HVA), which are intra- and extracellular metabolites, respectively (Figure 4E). Interestingly, DOPAC levels were significantly increased at 3 months (42%, Figure 4B), even though dopamine levels were modestly decreased. DOPAC is a catabolite of cytosolic (non-vesicular) dopamine (Figure 4E). Upregulation of DOPAC suggests cytosolic dopamine accumulation,^36^ which is known to be toxic. DOPAC accumulation is seen in both familial and sporadic models of PD.^37^ 3-Methoxytyramine (3-MT) is a dopamine metabolite, formed by direct catabolism of unused dopamine in the synaptic cleft by COMT^32^ (Figure 4E). 3-MT levels were significantly lower in auxilin KO mice (Figure 4C), suggestive of decreased dopamine release^38^ and reflective of the downregulation of COMT seen in the proteomics data (Figures 3E and 3F). Both DOPAC and 3-MT are metabolized further to HVA outside the DA termini (Figure 4E), whose level did not change at 3 months in auxilin KO mice (Figure 4D). This is possibly due to a balancing out of HVA levels attained by increased DOPAC and decreased 3-MT levels. At 9 months when motor abnormalities are apparent, both 3-MT and HVA levels were significantly decreased in auxilin KO mice (Figures 4C and 4D), which is also the case in PD patients. Indeed, decreased HVA levels have also been observed in the cerebrospinal fluid of patients with auxilin mutations.^15^ DOPAC levels were unchanged at 9 months. Levels of the serotonin metabolite 5-hydroxyindoleacetic acid did not change (Figure S7B). Overall, assessment of dopamine and its metabolites in dorsal striatum support the premise that dopamine homeostasis is altered in auxilin KO mice.

### Dopamine reuptake is dysfunctional in young auxilin KO mice

Extracellular dopamine in the striatum is pumped back into DA axons by the DAT. Therefore, DAT controls the level of presynaptically available dopamine and is a key regulator of dopamine compartmentalization and homeostasis.^39,40^ We evaluated extracellular dopamine clearance in the dorsal striatum of auxilin KO mice using fast scan cyclic voltammetry (FSCV) *in vivo*. The SNpc was stimulated using a paradigm that drives burst firing by nigral DA neurons. This causes dopamine buildup in the extracellular space at levels sufficient to saturate DAT and to be detected by the carbon-fiber electrode placed in the dorsal striatum^41^ (Figures 4F and S8A). Figures 4G and S8B depict the time course of evoked dopamine release and its clearance in the dorsal striatum, along with the characteristic background-subtracted voltammogram at the maximum oxidation peak, for WT and auxilin KO mice. Surprisingly, evoked dopamine release was not significantly different between WT and auxilin KO mice (Figure 4H). However, dopamine reuptake kinetics as measured by the time taken to clear 50% of the dopamine from its peak levels (t_1/2_) was significantly delayed in auxilin KO mice (Figure 4I), suggesting a pronounced deficit in DAT function.

To further analyze dopamine reuptake kinetics, we used a computational model^42^ to fit averaged FSCV traces from WT and auxilin KO mice. We found that the wider dopamine peak from auxilin KO mice can be closely fit by a ~73% reduction in dopamine reuptake (parameter $V_{m}$) and a ~54% reduction in dopamine release per electrical pulse (parameter ${DA}_{P}$) compared with WT mice ($V_{m}=2.0\;\mu \text{M}/\text{s}$ in auxilin KO vs. 7.4 μM/s in WT, ${DA}_{P}=0.31\;\mu \text{M}/\text{mA}$ in auxilin KO vs. 0.67 μM/mA in WT) (Figures 4J and S8C). While dopamine reuptake deficiency seen in the computational model was consistent with our FSCV recordings in auxilin KO, the decrease in dopamine release deviated from FSCV observations (Figure 4H). However, decreased neurotransmitter release is expected in auxilin KO mice, as these animals have previously been shown to have SV recycling defects.^23^ Our neurochemical analyses show decreased levels of the extracellular dopamine metabolite 3-MT (Figure 4C) in auxilin KO mice, which also indicate dopamine release defects. Thus, it appears that a larger decrease in dopamine reuptake is masking reduced dopamine release and can account for the greater variability and minimal difference in total evoked dopamine release observed between WT and auxilin KO mice in the FSCV recordings (Figure 4H).

### DAT deformities are present inthe dorsolateral striatum of auxilin KO mice

**Figure 5.**
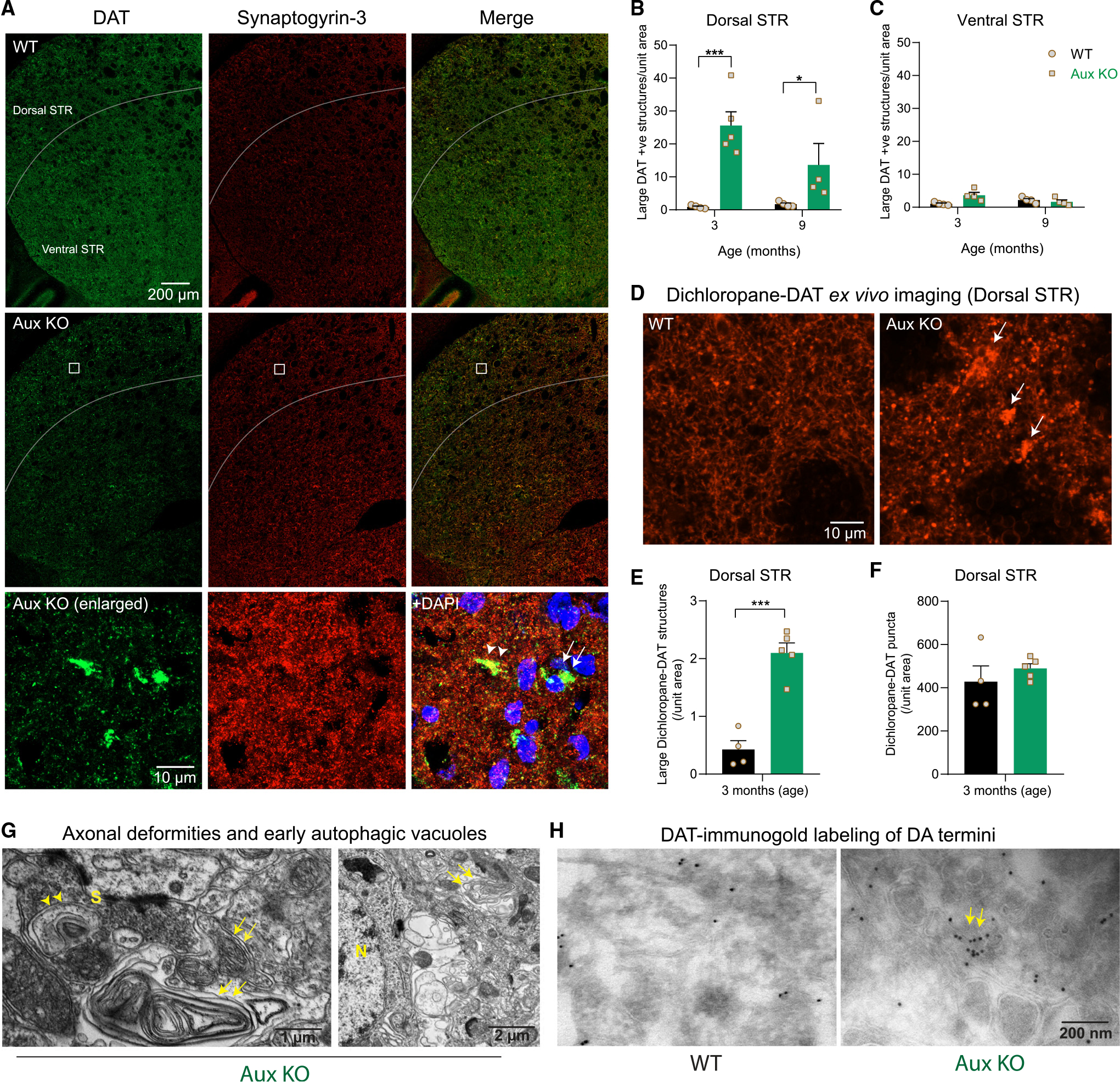
DAT^+^ axonal deformities in the dorsal striatum of auxilin KO mice (A) Representative images of dorsal and ventral striatum (STR) in WT and Aux KO mice (age 3 months), immunostained for DAT and synaptogyrin-3. Note large DAT^+^ structures in the dorsal STR of Aux KO mice that are absent in the ventral STR. Scale bar, 200 μm. These DAT^+^ structures were juxtaposed to presynapses (bottom row) as seen by colocalization with synaptogyrin-3 (Aux KO, enlarged, arrowhead), as well as in the soma marked by DAPI staining (Aux KO, enlarged, arrow). Scale bar, 10 μm. (B) Number of DAT^+^ structures/unit area in the dorsal striatum. (C) Note the absence of DAT^+^ structures in the ventral striatum. (D) Representative images of *ex vivo* staining of dichloropane-rhodamine red-X in the dorsal striatum of WT and Aux KO mice (age 3 months). DA axonal projections and presynaptic sites appear as small puncta, whereas axonal deformities appear as large dichloropane-DAT^+^ structures (arrows). Scale bar, 10 μm. (E) Number of large dichloropane-bound DAT^+^ structures/unit area in the dorsal striatum, which were significantly higher in Aux KO mice. (F) Number of small dichloropane-bound DAT^+^ puncta was not altered in Aux KO dorsal striatum in comparison with WT. (G) EM of dorsal striatum of Aux KO mice (age 3 months) showing large axonal whirl-like deformities (arrows), which were present ubiquitously, closer to both synaptic terminals (S) and soma (N, nuclei). Early autophagic vacuole-like structures were also seen in dorsal striatum (arrowheads), closer to axonal whirls. Scale bars, 1 μm and 2 μm. (H) DAT-immunogold labeling of dorsal striatum that mark only DA axonal termini showed dispersed labeling in WT. In Aux KO mice, DAT-immunogold clusters were seen in the dorsal striatum (arrows). Age 3 months. Scale bar, 200 nm. Data are presented as mean ± SEM. *p < 0.05, ***p < 0.001.

To visualize DAT in auxilin KO mice, we performed immunohistochemistry for DAT, co-labeling with the presynaptic SV protein synaptogyrin-3 in dorsal striatum (Figure 5A). We found large DAT^+^ structures (6–8 μm) in the dorsal striatum but not in the ventral striatum of auxilin KO brains (Figures 5A–5C and S9B), similar to what have been described in synaptojanin-1 knockin mice.^9^ These structures were absent in WT but ubiquitous in the dorsolateral striatum of auxilin KO mice (Figures 5 and S9A), localizing both with presynaptic sites (Figure 5A, enlarged, arrowheads) and closer to the soma (as marked by DAPI; Figure 5A [enlarged, arrows] and Figure S9C). DAT^+^ structures were observed in auxilin KO mice at both 3 and 9 months of age, and were significantly higher (~40%) at the earlier time point (Figure 5B). Synaptogyrin-3, a known interactor of DAT and marker of presynaptic termini, did not exhibit a change in distribution or expression level (Figures 5A and S10F). Glutamatergic and GABAergic termini did not exhibit such structures (Figures S10A–S10E). As DAT is typically localized to DA axonal projections,^43^ these observations suggest that the large DAT^+^ structures seen at the dorsal striatum of auxilin KO may be DA axonal membrane deformities.

To confirm that the DAT^+^ structures are membrane bound and surface accessible, we performed *ex vivo* imaging using the membrane DAT ligand dichloropane, which binds to cell surface DAT preferentially from the extracellular side. Dichloropane was conjugated with rhodamine red-X to obtain the dichloropane-rhodamine red-X probe.^44^ Fresh striatal slices were incubated in artificial cerebrospinal fluid (ACSF) containing dichloropane probe and imaged for membrane-bound DAT. Auxilin KO mice revealed large dichloropane-DAT^+^ structures (Figures 5D and 5E, arrows) similar to those in the dorsal striatum of fixed brains, suggesting that the DAT structures are membrane accessible. The number of small dichloropane-DAT^+^ puncta that represent DA terminal varicosities in the dorsal striatum were not altered in auxilin KO mice at 3 months of age (Figures 5D–5F). This also suggests that there is no absolute loss of DAT in the dorsal striatum but a mislocalization of DAT to axonal membrane deformities. Next, we performed an ultrastructural evaluation of dorsal striatum by electron microscopy (EM), which revealed multilayered axonal whirls in auxilin KO brains (Figures 5G and S11A, arrows). Additionally, there were early autophagic vacuole-like structures close to these axonal deformities (Figure 5G, arrowheads). We performed immunogold labeling of DAT in the dorsal striatum, which revealed a uniform distribution of DAT-immunogold particles in WT, denoting DA axonal projections (Figure 5H). In contrast, DAT-immunogold clusters were observed in auxilin KO mice (Figures 5H and S11B, arrows), indicative of axonal membrane deformities of DA projections. Collectively, these observations suggest that DAT is mistrafficked and trapped in large axonal deformities, which hinder DAT function in dopamine reuptake.

### Dorsal striatum of auxilin KO mice shows minimal endocytic alterations

Yim et al.^23^ have previously noted increased clathrin in whole-brain homogenates of auxilin KO pups. We saw a similar increase of clathrin levels in whole-brain homogenates of 3- and 9-month-old auxilin KO mice (Figures S5D and S5E). Endophilin-A1 levels were mildly altered (Figures S5F and S5G). We evaluated the distribution pattern of these endocytic partners of auxilin in the dorsal striatum. We immunostained for clathrin and saw that neither clathrin intensity nor its distribution pattern was altered in young auxilin KO mice (Figures S12A and S12B). Hsc70, the chaperone partner of auxilin, and endophilin-A1, another key endocytic protein required for uncoating, also did not change significantly (Figures S12A–S12E) in auxilin KO mice at 3 months. These observations remained true even at 9 months, except for endophilin-A1 which was significantly upregulated in auxilin KO mice (Figures S12A and S12C). We assessed the interaction of clathrin with Hsc70 and endophilin-A1 by their colocalization, which was not altered in auxilin KO mice (Figures S12A, S12F, and S12G). Overall, there were no major changes in endocytic protein composition and distribution in the dorsal striatum of young auxilin KO mice.

**Figure 6.**
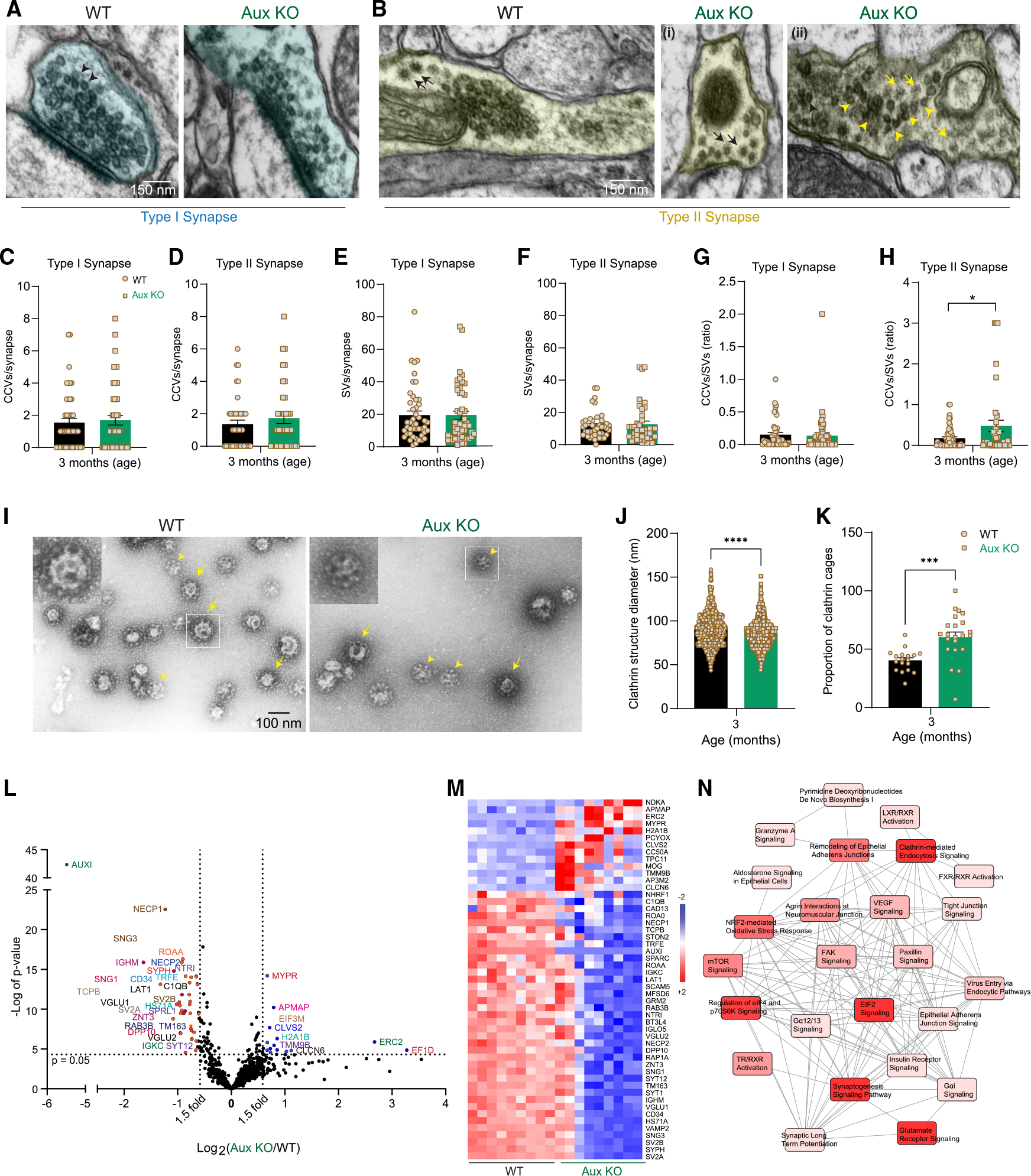
EM and proteomics revealed imbalance in CCV/SV ratio and SVs with variable membrane composition in auxilin KO mice (A) Representative EM image of a type I excitatory presynapse with SVs (arrowheads) from dorsal striatum of WT and Aux KO mice (age 3 months). Scale bar, 150 μm. (B) Representative EM image of a type II inhibitory presynapse in the dorsal striatum of WT and Aux KO mice with SVs (arrows) and CCVs (arrows). (i) Aux KO presynapse showing an increase accumulation of CCVs. (ii) Another Aux KO synapse showing CCVs or clathrin cage accumulation (arrows), as well as SV clusters (arrowheads). Scale bar, 150 μm. (C) Number of CCVs in type I synapses. (D) Number of CCVs in type II synapses. (E) Number of SVs in type I synapses. (F) Number of SVs in type II synapses. (G) The CCV/SV ratio in type I synapses of dorsal striatum. (H) The CCV/SV ratio in type II synapses of dorsal striatum. (I) Representative EM images of CCV preparation showing CCVs (arrows) and empty clathrin cages (arrowheads) in WT and Aux KO mice. Scale bar, 100 nm. (J) Diameter of clathrin structures (CCVs + clathrin cages). (K) Proportion of clathrin cages in WT and Aux KO mice. (L) Volcano plot of CCV proteome of Aux KO in comparison with WT mice (n=14mice/experiment,3experiments/genotype). Proteins that were changed 1.5-fold (vertical dotted lines) with a p value of 0.05 (Student’s t test) or less (horizontal dotted line) were considered as significantly changed. (M) Heatmap of significantly changed proteins in Aux KO in comparison with WT mice for each experiment. Red indicates increased expression (+2) and blue indicates decreased expression (−2). (N) Pathways that are significantly affected (p<0.05) in Aux KO mice due to CCV proteome changes, and their overlap. Pathways depicted in intense red are highly affected, whereas those in light red are moderately affected. Data are presented as mean± SEM. *p < 0.05, ***p < 0.001, ****p < 0.0001.

To confirm our immunohistochemistry findings, we performed EM of the dorsal striatum of 3-month-old WT and auxilin KO mice and quantitated the number of CCVs (Figure 6B, arrows) and SVs (Figures 6A and 6B, arrowheads) per synapse. We quantified these organelles in both asymmetric or type I synapses which are predominantly glutamatergic (Figure 6A), and symmetric or type II synapses which are known to be DA or GABAergic in the dorsal striatum^45^ (Figure 6B). The number of CCVs in type I synapses showed a moderate increase in auxilin KO mice (~10%, Figure 6C). This was pronounced in type II synapses of auxilin KO mice (~27%, Figure 6C). No notable difference between WT and auxilin KO mice was found in SV number in both type I and type II synapses (Figures 6E and 6F). The cumulative effect of this was seen in the CCV/SV ratio, which showed a modest but significant increase only in type II synapses (Figures 6F and 6G). It is worth noting that the distribution of CCVs and SVs was variable within the type II synapses of auxilin KO mice (compare Figures 6Bi and 6Bii). Overall, these results are consistent with our immunohistochemistry, which did not show notable difference in clathrin distribution (Figures S12A and S12B), and previous findings on cerebellar presynapses of auxilin KO mice.^23^

### CCV proteomics in auxilin KO mice suggest SV sorting defects

An increase in cytoplasmic dopamine levels (Figure 4) suggests lack of functional SVs and/or improper neurotransmitter sequestration into SVs. To understand the impact of loss of auxilin on SV sorting and composition, we performed EM and proteomic analysis of CCVs purified from brains of WT and auxilin KO mice (age 3 months).^46^ EM of the CCV preparations revealed that they are pure, contain both CCVs (arrows) and clathrin cages (arrowheads), and lack SVs (Figure 6I).^47^ Auxilin KO mice displayed clathrin structures (CCVs + clathrin cages), which were significantly smaller in size compared with WT mice (Figure 6J). This is in part because there was a larger proportion of clathrin cages in auxilin KO mice (Figure 6K), consistent with previously published findings.^23^

LFQ-MS of CCVs revealed 891 proteins common to three independent experiments, 49 of which were significantly changed, with the majority being downregulated (38 downregulated, 13 upregulated, Table S3). Strikingly, all the proteins that exhibit decreased levels were SV transmembrane proteins,^48,49^ such as SNG1 and SNG2, SYP, SYT1 and SYT12, and SV2A and SV2B (Figures 6J and 6K). VGLUT1 and VGLUT2, vesicular transporters for the excitatory neurotransmitter glutamate were decreased (Figures 6I–6M). Vesicular zinc transporters such as ZNT3 and TM163 were also decreased (Figures 6I–6M). By extension, this suggests that vesicular monoamine transporter-2 (VMAT2) may also be decreased, which was not detected by LFQ-MS due to its low abundance.^49^ IPA analysis of the CCV proteomics revealed dysregulation in the CME pathway in auxilin KO mice (Figure 6N).

To rule out the possibility that the downregulation of certain SV transmembrane proteins seen in the auxilin KO CCV proteomics was due to the presence of clathrin cages, we compared our CCV proteomics data with published SV proteomics data.^48^ We found that the levels of synapsins, SCAMPs, syntaxins, SNAPs, and several others which are categorized as SV trafficking proteins were unchanged. Endocytic proteins such as dynamins, flotilins, RAB proteins, endophilin-A1, and synaptojanin-1, which are peripherally associated with the SV membrane, were also unchanged in auxilin KO CCVs compared with WT CCVs. These observations indicate that the decrease in certain SV transmembrane proteins in auxilin KO mice is likely not an artifact. Overall, these results suggest SV sorting defects congruent with the initial findings in Yim et al., who found synaptophysin-pHluorin stranded on the membrane^23^ and recently proposed roles for auxilin in endocytic proofreading.^50^ These data indicate that uncoating of CCVs in auxilin KO mice by GAK as well as alternative ways of SV formation^51^ would result in SVs with an improper protein stoichiometry.

### Auxilin KO mice show increased presynaptic autophagy

**Figure 7.**
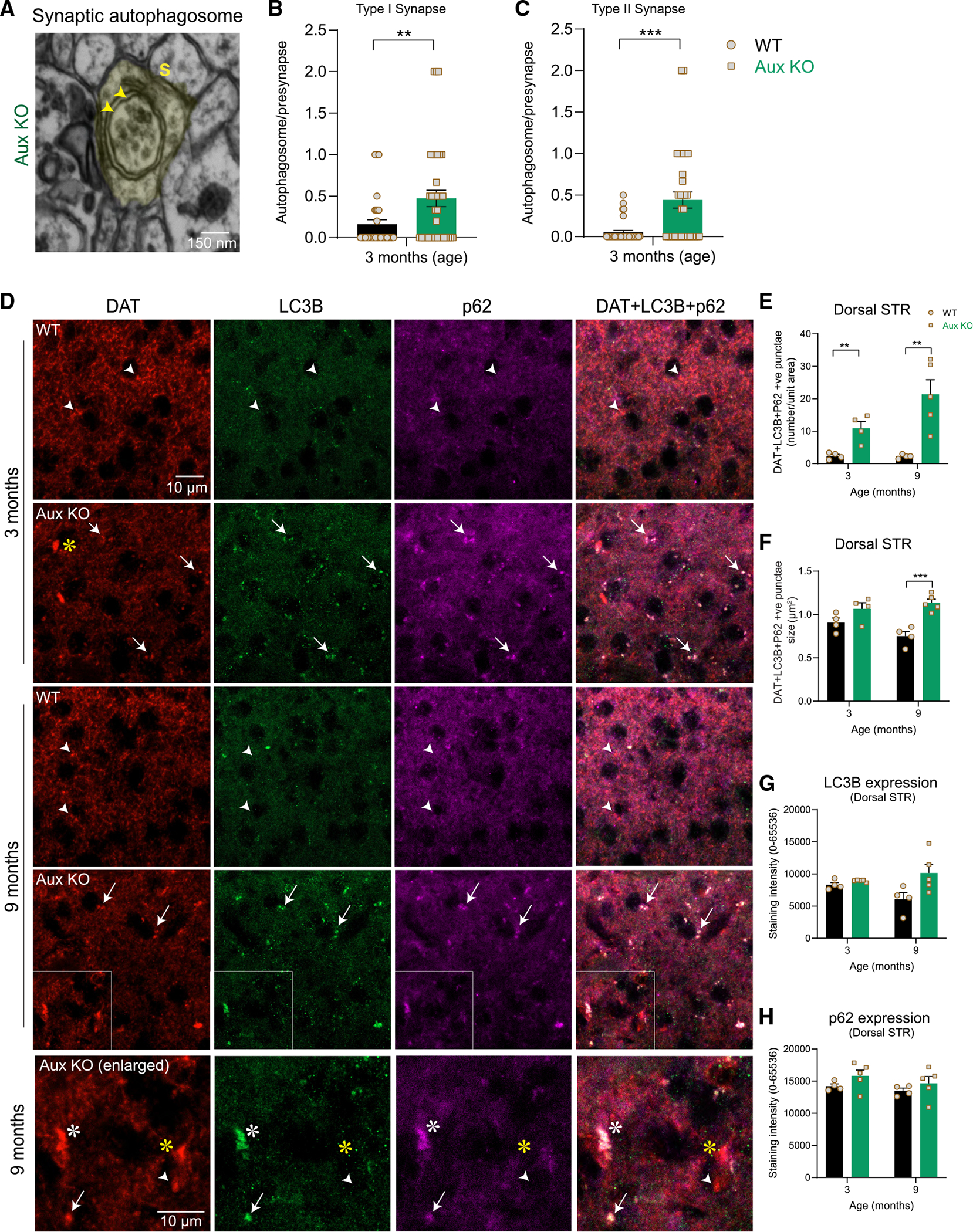
Synaptic autophagy is enhanced in the dorsal striatum of auxilin KO mice (A) Representative EM image of a type II synaptic terminal (S) in the dorsal striatum of Aux KO mice showing double-membrane autophagosomes containing CCVs/clathrin cages/missorted SVs (arrows). Scale bar, 150 μm. (B) Autophagosomes per presynaptic terminal in type I synapses. (C) Autophagosomes per presynaptic terminal in type II synapses. (D) Representative images of dorsal striatum immunostained for DA termini marker DAT (red) and autophagosome markers LC3B (green) and p62 (magenta). Note high number of DAT^+^ DA termini colocalizing with LC3B and p62 (arrows, DAT + LC3B + p62: white) in Aux KO mice, whereas DA termini in WT mice did not show such colocalization (arrowheads). In Aux KO mice, a few large DAT^+^ structures (axonal whirls) were positive for LC3B and p62 (Aux KO, enlarged, white asterisks) while others were not (Aux KO, enlarged, yellow asterisks). Scale bar, 10 μm. (E) Number of DAT^+^ termini colocalizing with LC3B and p62 was significantly higher in Aux KO mice. (F) Size of DAT + LC3B + p62-positive puncta was larger in 9-month-old Aux KO compared with WT mice. (G) LC3B expression in dorsal striatum did not show any significant difference between WT and Aux KO mice. (H) p62 expression showed a trend toward higher expression in Aux KO mice, although not significant. Data are presented as mean ± SEM. **p < 0.01, ***p < 0.001.

To investigate whether CCVs, clathrin cages, and missorted SVs are cleared by autophagy,^52,53^ we examined the electron micrographs of WT and auxilin KO dorsal striatal presynapses for double-membrane synaptic autophagosomes (Figures 6Bii and 7A). The number of autophagosomes was significantly higher in both type I and type II synapses in auxilin KO compared with WT mice (Figures 7B and 7C). Furthermore, we found several examples of autophagosomes, in the type II synapses, containing CCVs and SVs as their cargo (Figure 7A, arrowheads). To validate the EM observations we performed immunohistochemistry in the dorsal striatum, examining autophagosome markers LC3B and p62 and their colocalization with DAT (Figure 7D: DAT, green; LC3B, red; p62, magenta; DAT + LC3B + p62, white). We noted a significant increase in several DAT^+^ DA synaptic boutons, which are also positive for LC3B and p62 in auxilin KO mice (Figure 7D, Aux KO, 3 and 9 months, arrows; Figures 7E and 7F). The number and size of the DA boutons that are positive for LC3B and p62 were relatively higher at 9 months compared with 3-month-old auxilin KO mice (Figures 7E and 7F), suggesting worsening pathology with age. Although there was a trend toward an increase in overall LC3B and p62 expression in dorsal striatum, it was not significant (Figures 7D, 7G, and 7H). We confirmed LC3 and p62 expression by western blotting of total brain and synaptosome homogenates (Figures S5H–S5O). Not all large DAT^+^ axonal deformities (Figure 5) were positive for autophagosome markers (Figure 7D, Aux KO, white vs. yellow asterisks). However, we observed LC3B^+^ and p62^+^ puncta in close vicinity to a subset of large DAT^+^ structures just as we saw in our EM analysis (Figure 5G), suggesting a possible role of autophagic overload in the formation of axonal deformities. Overall, these results suggest that accumulated CCVs, missorted SVs and other endocytic intermediates are cleared by an increase in presynaptic macroautophagy in auxilin KO mice.

## DISCUSSION

Recent advancements in PD genetics strongly point to disruptions in clathrin uncoating and SV endocytosis as important for the pathogenesis of PD.^2,21,54^ Here, we show that KO of the clathrin-uncoating chaperone auxilin in mice replicates all the key features of PD—age-dependent α-synuclein pathology, selective dopaminergic neuron loss, and gliosis—resulting in motor deficits. Crucially, treatment with L-DOPA can rescue the motor deficits of aged auxilin KO mice. Thus, auxilin KO mice show both construct and face validity for PD. Compared with other mouse models of PD, including α-synuclein transgenics^55,56^ and endocytic mutants,^9,14^ auxilin KO mice recapitulate a complete repertoire of phenotypes. We took advantage of this feature and the fact that auxilin has a defined function in clathrin uncoating to elucidate the underlying mechanisms. We show that auxilin deficiency leads to neurodegeneration through three distinct but overlapping mechanisms in nigrostriatal DA termini: (1) toxic accumulation of cytoplasmic dopamine due to imbalance in CCV/SV ratio and defective sorting of SVs; (2) mistrafficking of DAT that traps the protein in axonal membrane whirls, leading to defective dopamine reuptake; and (3) synaptic autophagy overload. Collectively, these mechanisms lead to dopamine dyshomeostasis, a trigger of neurodegeneration in PD.

### Accumulation of cytoplasmic dopamine

Dopamine is typically sequestered into SVs via VMAT2 to avoid auto-oxidation. Dopamine that accumulates in the cytoplasm is oxidized predominantly to DOPAL and subsequently catabolized to DOPAC. Thus, the elevated DOPAC levels observed in the dorsal striatum of presymptomatic auxilin KO mice is an indirect measure of cytoplasmic dopamine accumulation^36^ and increased conversion to DOPAL, a mediator of dopamine-related toxicity in PD.^37^ The accumulation of DOPAC is likely due to two factors: an imbalance in the CCV/SV ratio and SVs with improper composition. Owing to slowed CME,^23^ auxilin KO neurons need to utilize alternative endocytic pathways to maintain SV pools, which are less efficient and stringent in protein sorting, leading to SVs of variable protein composition.^51^ Proteomic analysis of CCVs from auxilin KO brains confirmed this tenet and revealed a smaller copy number of integral SV membrane proteins. Thus, loss of auxilin is likely to lead to fewer functional SVs available for neurotransmitter filling and release. This is also supported by our neurochemical analysis of the extracellular dopamine metabolite3-MT which decreases, indicative of dopamine release defects.^38^ The FSCV-based computational model also predicted dopamine release defects, suggesting defective SV sequestration of dopamine in auxilin KO mice, which is seen in patients with PD.^57^ Although our CCV proteomic analysis was not sufficiently sensitive to detect VMAT2, it could be downregulated in auxilin KO CCVs considering the decrease of two other key vesicular neurotransmitter transporters, VGLUT1 and VGLUT2. VMAT2-deficient mice also exhibit cytosolic dopamine accumulation and develop PD phenotypes.^26^ Previous studies have shown that DOPAL-modified α-synuclein oligomers form pores in SVs that cause increased DA leakage into the cytoplasm.^58^ While SV sorting deficits probably occur in all synapses, leading to neurotransmitter packaging defects, the properties of dopamine catabolites such as DOPAL are likely to render DA synapses vulnerable.

### Dopamine reuptake dysfunction and DAT mislocalization in membrane deformities

Extracellular dopamine in the synaptic cleft is cleared by reuptake into presynapses through DAT and/or enzymatic degradation to 3-MT by COMT. In dorsal striatum, reuptake by DAT plays a major role in clearing extracellular dopamine whereas COMT has a negligible role.^32,59^ Hence, nigrostriatal DA presynapses depend heavily on DAT-mediated dopamine reuptake to replenish their readily releasable neurotransmitter pool. A significant delay in clearing evoked dopamine from the dorsal striatum *in vivo* that was modeled *in silico* along with presence of large DAT^+^ deformities both in fixed tissue and *ex vivo* clearly indicate that DAT is dysfunctional in auxilin KO mice. This appears to be a defining feature of DA neurodegeneration in auxilin KO mice. Dopamine reuptake dysfunction for an extended period may lead to striatal dopamine loss, as seen in DAT KO mice.^40^

Live slice imaging of rhodamine-tagged dichloropane, which binds to plasma membrane DAT, suggests that there is no absolute loss of dorsal striatal DAT in auxilin KO mice but that DAT is trapped in the axonal membrane deformities. This was confirmed by proteomics and DAT-immunogold labeling. No change in DAT levels was seen in LFQ-MS. Similar DAT^+^ membrane whirls were seen in synaptojanin-1 mutants,^9^ which showed exacerbated pathology when crossed with auxilin KO mice,^60^ whereas synaptojanin-1 overexpression rescued the pathology in Drosophila carrying auxilin mutation.^61^ Other evidence of axonal damage comes from our proteomic findings where neurofilament proteins that maintain axonal integrity were altered, including an increase in NF-L. Together, these observations suggest that the dopamine reuptake decrement seen in auxilin KO mice occurs principally because of DA axonal membrane deformities that trap DAT. In DA presynapses, membrane localization of DAT is dynamically regulated by endocytic trafficking and recycling. It remains to be determined whether auxilin participates in endocytic recycling of DAT in DA neurons, and this will be explored in the future.

### Synaptic autophagy overload

Owing to the higher turnover of synaptic proteins, vesicles, and mitochondria in presynaptic sites, autophagosome biogenesis occurs at a higher rate in the distal axons than in the soma.^62^ Because of limited lysosomal activity, synaptic termini depend on retrograde microtubule-based axonal transport of autophagosomes toward the lysosome-rich soma for degradation. Tonic activity of DA neurons is likely to keep basal autophagy rates high, and the requirement to transport autophagosomes long distances via extensive arborization makes DA axons vulnerable to additional autophagic burden. We show an increased number of autophagosomes in dorsal striatal DA termini of auxilin KO mice by immunostaining and ultrastructural evaluation, which also revealed several examples of autophagosomes containing CCVs, clathrin cages, and SVs. We find evidence for increased mTOR signaling and activation of autophagic pathways in the synaptosomal proteomic data (9-month), supporting elevated synaptic autophagy in auxilin KO mice. Rapamycin-induced enhancement of autophagy in DA presynapses of mice striatal slices has been shown to sequester SVs and decrease evoked dopamine release.^63^ A similar event in auxilin KO synapses might worsen cytosolic dopamine accumulation. Enhanced synaptic autophagy to clear missorted vesicles, as well as the products of toxic dopamine oxidation, could burden DA projections with autophagic vacuoles. Autophagosome accumulation, impaired retrograde transport, and abnormal axonal deformities in DA axons have been previously seen in neurons from patients with PD and Alzheimer’s disease.^64–66^ EM revealed some of the autophagic vacuoles near the whirl-like axonal deformity in auxilin KO striatum. Although we presently do not understand the relationship between these two structures, DA axonal deformities observed in auxilin KO mice may be a result of autophagic overload in DA termini.

In conclusion, our findings indicate that pathology of PD mediated by auxilin deficiency begins with a disruption of CME, which leads to fewer functional SVs for neurotransmitter filling. While these deficits occur at all synapses, they appear to have a particularly detrimental effect at nigrostriatal DA synapses due to the toxicity of cytosolic dopamine and DAT reuptake alterations. Thus, investigating auxilin loss of function has also enhanced our understanding of the mechanisms for DA vulnerability in PD.

### Limitations of the study

Our characterization of auxilin KO mice for PD-like features is thorough and utilized unbiased proteomics. While we chose to follow up on the major implicated pathways, validating individual proteins is also important. Although we have strong evidence for the accumulation of intracellular dopamine metabolite DOPAC and evidence for dopamine release defects in the dorsal striatum, these are indirect measures of cytosolic dopamine accumulation and its toxicity. Methods that directly measure cytosolic or oxidized dopamine are hard to come by for murine models. We have clear evidence for an increase in synaptic autophagy which appears to be a cellular response to clear missorted SVs and CCVs. Additional assays are required to confirm this and its contribution to nigrostriatal neurodegeneration in auxilin-linked PD. Finally, the mechanisms leading to α-synuclein aggregation in auxilin KO mice need to be investigated.

## STAR★METHODS

### RESOURCE AVAILABILITY

#### Lead contact

Further information and requests for resources and reagents should be directed to and will be fulfilled by the lead contact, Sreeganga S. Chandra (sreeganga.chandra@yale.edu).

#### Material availability

This study did not generate new unique reagents.

#### Data and code availability

- The raw mass spectrometery/proteomics data have been deposited in the publicly available PRIDE depository and are publicly available as of the date of publication. Accession numbers are listed in the key resources table. Catalog numbers, RRIDs, and DOIs are listed in the key resources table.
- This paper does not report original code.
- Any additional information required to reanalyze the data reported in this paper is available from the lead contact upon request.

### EXPERIMENTAL MODEL AND SUBJECT DETAILS

#### Animals

Auxilin KO mice (congenic B6.-*Dnajc6^tm1Legr^*)^23^ were bred to C57BL6/J mice to make them congenic. Auxilin homozygous KO mice were compared to WT C57BL6/J from Jackson Laboratories, Maine. Mice of both sexes and age ranging from 3–15 months were used for behavior experiments, and 3 and/or 9 months for all other experiments. Mice were maintained on a 12-hour light-dark cycle with access to standard chow ad libitum. All animal experiments were executed in accordance with the National Institutes of Health guidelines for the Care and Use of Laboratory Animals and with the approval of the Yale University Institutional Animal Care and Use Committee.

### METHOD DETAILS

#### Motor behavior evaluation

WT and auxilin KO cohorts were examined longitudinally at 3, 6, 9, 12, and 15 months of age (n=12–16 mice/genotype, sex-balanced) in motor behavioral assays. For evaluation of overall locomotory capabilities, mice were allowed to explore an open field arena, which was videotaped to assess the distance travelled in 5 mins using Noldus Ethovision XT software. The number of fecal pellets excreted during open field behavior test was evaluated as a measure of anxiety. The balance beam test was used to assess motor coordination by evaluating the ability to walk straight on a narrow beam from a brightly lit end towards a dark and safe box (Video S1). Latency to traverse the beam and the number of times a mouse could perform this behavior in a minute were evaluated. Mice, when picked up by the tail and lowered towards the ground, extend their limbs reflexively in anticipation of contact. Mice with certain neurological diseases display hind limb clasping instead of extension. We performed this maneuver for 30 seconds and scored the hindlimb clasps (0: no clasp; 1: One hind limb clasp; 2: both the hind limbs clasp, Video S2) and noted total time clasping. The grip strength of the forelimbs and all the limbs was assessed by measuring the maximum force (g) exerted by the mouse in grasping specially designed pull bar assemblies attached to a grip strength meter (Columbus Instruments, Ohio, USA) in tension mode. A four-lane Rotarod was used to assess motor coordination and balance (Columbus Instruments, Ohio, USA). Mice were made to run for 300 secs on the rotating spindle of the Rotarod, which was accelerating from 4 to 40 rpm. Each mouse was subjected to three trials with a 30 min inter-trial recovery period. The average of the latency to fall and the rpm in these trials was used as a measure of motor performance. The procedure was repeated for four consecutive days in both WT and auxilin KO mice. We did not see a significant sex-based differences in all the behavior assays in auxilin KO mice and data from both sexes was collated.

#### L-DOPA treatment

A separate set of auxilin KO mice (n=7–8) at symptomatic age of 9 (n=5–6) to 12 months (n=2) with age-matched WTs (9 months, n=5) were treated with one dose of L-DOPA (intraperitoneal, 15mg/kg body weight, injected in 10ml/kg volume of 0.85% saline). One week before the L-DOPA injection (Pre-treatment), the mice were subjected to open field and balance beam behavior experiments, as described above. A week later, 20–25 minutes after L-DOPA injections (Post-treatment), open field and balance beam behavior assays were repeated to check if L-DOPA could improve motor behavior deficits of auxilin KO mice. Appropriate vehicle controls were used.^67^

#### Immunohistochemistry

WT and auxilin KO mice at 3 and 9 months of age (n=5–6/group; sex-balanced) were anaesthetized using isoflurane inhalation and perfused intracardially with 0.9 % heparinized saline followed by chilled 4 % paraformaldehyde (PFA) in 0.1 M phosphate buffer (PB). The brains were post-fixed in the same buffer for 48 hours and cryoprotected in increasing grades of buffered sucrose (15 and 30 %, prepared in 0.1 M PB), at 4°C, and stored at −80°C until sectioning. Serial sections of the brains (30 μm thick) were performed coronally using a cryostat (Leica CM1850, Germany), collected on gelatinized slides, and stored at −20°C. Every sixth nigral section was subjected to immunoperoxidase staining and every 10^th^ striatal section was used for immunofluorescence staining as per our earlier protocol.^27^ Briefly, for immunoperoxidase staining, endogenous peroxidase quenching was performed using 0.1 % H_2_O_2_ in 70 % methanol (30 mins), followed by blocking using 3 % bovine serum albumin (BSA) (2 hours) at room temperature (RT). Sections were then incubated at 4°C with TH primary antibody (1:500, overnight) followed by biotin-conjugated secondary antibody at RT (1:200; 3–4 hours, Vector Laboratories, PK-6101). Tertiary labeling was performed with the avidin-biotin complex solution at RT (1:100; 3–4 hours, Vector Laboratories, PK-6101). Staining was visualized using 3,3′-diaminobenzidine (Fluka, 32750) as a chromogen in a solution of 0.1 M acetate imidazole buffer (pH 7.4) and H_2_O_2_ (0.1 %). For immunofluorescence staining, sections were incubated in 0.5 % Triton-X-100 (15 mins), followed by incubation in 0.3 M glycine (20 mins). Blocking was performed using 3% goat serum, followed by overnight incubation (4°C) in primary antibodies. Sections were then incubated in Alexa-conjugated secondaries (Thermo Fisher Scientific, USA) for 3–4 hours, followed by coverslip mounting using an antifade mounting medium with (H-1000, Vectashield) or without DAPI (H-1200, Vectashield). Coverslips were sealed using nail polish. 1X PBS with 0.1 % Triton-X-100 was used as both washing and dilution buffer for both immunoperoxidase and immunofluorescence staining, except for pSer129-α-Syn where 1X Tris buffer saline was used. Below is the list of antibodies used and their dilutions.

**Table:** 

Antibody	Dilution	Manufacturer RRID

Rabbit Anti-TH	1:500	Millipore (AB152) AB_390204
Mouse Anti-TH	1:500	Synaptic Systems (213211) AB_2636901
Rabbit Anti-Iba1	1:300	Wako Chemicals (019-19741) AB_839504
Guinea Pig Anti-GFAP	1:400	Synaptic Systems (173004) AB_10641162
Guinea Pig Anti-DAT	1:300	Synaptic Systems (284005) AB_2620019
Rabbit Anti-α-synuclein (phospho S129)	1:800	Abcam (ab51253) AB_869973
Mouse Anti-Clathrin light chain	1:200	Synaptic Systems (113011) AB_887706
Rabbit Anti-Synaptogyrin 3	1:200	Synaptic Systems (103303) AB_2619753
Rabbit Anti-VGAT	1:500	Synaptic Systems (131002) AB_887871
Guinea Pig Anti-VGLUT2	1:500	Synaptic Systems (135418) AB_2864786
Rat Anti-Hsc70	1:100	Enzo (ADI-SPA-815-D) AB_2039279
Rabbit Anti-Endophilin-A1	1:200	Synaptic Systems (159002) AB_887757
Guinea Pig Anti-NeuN	1:500	Millipore (ABN90) AB_11205592
Rabbit Anti-LC3B	1:200	Abcam (192890) AB_2827794
Mouse Anti-p62/SQSTM1	1:200	Novus (H00008878-M01) AB_548364

#### Unbiased stereology

The SNpc was delineated on every 6^th^ TH^+^ midbrain section^68^ using a 10X objective of a brightfield microscope equipped with StereoInvestigator (Software Version 8.1, Micro-brightfield Inc., Colchester, USA). The stereological quantification of TH^+^ DA neurons was performed using the optical fractionator probe of the StereoInvestigator.^27^ The neurons were counted using 40X objective, with a regular grid interval of 22,500 μm^2^ (x=150 μm, y=150 μm) and a counting frame size of 3600 μm^2^ (x=60 μm, y=60 μm). The mounted thickness was identified to be around 22.5 μm, which was also determined at every fifth counting site. A guard zone of 3.5 μm was implied on either side, thus providing 15 μm of z-dimension to the optical dissector. The quantification began at the first anterior appearance of TH^+^ neurons in SNpc and VTA to the caudal most part in each hemisphere^68^ separately, which was later summed to derive total numbers. Counting was performed blinded to the genotype.

#### Microscopy and image analysis

Fluorescent images were acquired using a laser scanning confocal microscope (LSM 800, Zeiss) with a 20X or 40X or 63X objectives, and using fluorescence slide scanner (VS200, Olympus) at 40X objective (to image NeuN^+^ DA neurons). Appropriate Z-depth was used. All the images were blinded for genotype and age before subjecting to analysis using FIJI software from National Institute of Health (NIH). After performing sum intensity projection, the expression intensity was measured on an 8-bit or 16-bit image as the mean gray value on a scale of 0–255 or 0–65536, respectively. For counting Iba1^+^ microglial cells, images were thresholded using the ‘otsu’ algorithm and the cells larger than 75-pixel units for a given image were counted using the ‘analyze particles” function. Similar method was used to count immunofluorescence labeled TH^+^ and NeuN^+^ cells using size threshold of 25 μm^2^ and above. GFAP^+^ astroglial cells were counted manually using the ‘cell counter’ function. For counting DAT^+^ structures, images were thresholded using ‘triangle’ algorithm, followed by ‘analyse particles’ function. All the structures of size 5 μ^2^ and above and the circularity between 0.3 to 1 were counted. DAT+LC3B+p62 punctae which looked white when colocalized were counted manually using ‘cell counter’ function, which was further validated on thresholded images using ‘triangle’ algorithm, followed by ‘analyse particles’ function (size: 0.5 μ^2^, circularity: 0.0 to 1). Colocalization analysis was performed using “Coloc 2” function. SNpc, VTA and SNpr were demarcated as per Fu *et al*.^68^, colabeling with TH-immunostaining. Dorsal and ventral striata were demarcated as per Paxinos and Franklin, 2008.^69^

#### Western blotting

Western blotting was performed as per our standard protocol.^70^

**Table:** 

Antibody	Dilution	Manufacturer RRID

Rabbit Anti-auxilin	1:400	De Camilli lab, Yale University NA
Mouse Anti-β-actin	1:1000	Genetex (GTX629630) AB_2728646
Mouse Anti-synaptobrevin-2	1:10,000	Synaptic Systems (104211) AB_887811
Mouse Anti-clathrin light chain	1:5000	Synaptic Systems (113001) AB_887705
Mouse Anti-endophilin-A1	1:1000	Synaptic Systems (159002) AB_887757
Rabbit Anti-LC3B	1: 1000	Sigma-Aldrich (L7543) AB_796155
Mouse Anti-p62/SQSTM1	1: 1000	Novus (H00008878-M01) AB_548364

#### Proteomic analysis

Whole brain, whole brain synaptosomes and CCV samples were prepared from 3-month-old WT and auxilin KO mice. Brains from 3-month-old WT and auxilin KO mice (n=3/genotype) were homogenized in homogenization buffer (detergent-free 320 mM sucrose in 10 mM HEPES, pH 7.4 with protease and phosphatase inhibitors cocktail). Part of the homogenate was snap-frozen for whole brain proteomics. Rest of the homogenate was used to prepare synaptosomes as per our established protocol.^71^ Synaptosomes integrity was confirmed by EM and enrichment of synaptosome fractions were confirmed as per Gorenberg et al.^70^ before performing LFQ-MS. For CCVs sample preparation, brains from 14 pairs of WT and auxilin KO mice were pooled to obtain a single CCV fraction.^46,47^ Three independent purifications were performed and the resulting CCVs fractions were subjected to LFQ-MS. The purity of CCVs was confirmed by EM (Figure 7A).

LFQ-MS was performed at Yale Mass Spectrometry & Proteomics Resource of the W.M. Keck Foundation Biotechnology Resource Laboratory. Samples were analyzed in technical triplicates. The data were normalized to internal controls and total spectral counts. Proteins with two or more unique peptide counts were listed using UniProt nomenclature and included for further analysis. A 1.5-fold change and a p-value difference of <0.05 between WT and auxilin KO are considered as significant. Heat maps for significantly changed proteins were produced using Qlucore Omics Explorer. IPA (Qiagen) was used to determine the most significantly affected canonical pathways and their overlap.

#### High-performance liquid chromatography (HPLC)

Sex balanced, auxilin KO mice at 3 and 15 months of age with appropriate controls (n=8–12/genotype) were anesthetized using isoflurane inhalation. Mice were then sacrificed by cervical dislocation, and the brains were quickly removed and dissected for dorsal striatum, snap frozen, and stored at −80°C until further use. For HPLC, tissues were homogenized, using a handheld sonic tissue dismembrator, in 100–750 ul of 0.1M TCA containing 0.01M sodium acetate, 0.1mM EDTA, and 10.5 % methanol (pH 3.8). The samples were then spun in a microcentrifuge at 10,000 g for 20 minutes. Supernatant was removed for HPLC-ECD (electrochemical detection) analysis. HPLC was performed using a Kinetix 2.6um C18 column (4.6 × 100 mm, Phenomenex, Torrance, CA USA). The same buffer used for tissue homogenization is used as the HPLC mobile phase. Protein concentration was estimated using Pierce^™^ BCA Protein Assay Kit (Thermo Scientific). HPLC experiments were performed in the Vanderbilt University Neurochemistry Core. We did not notice a significant sex-based difference in HPLC results in auxilin KO mice.

#### Surgery and *in vivo* fast scanning cyclic voltammetry (FSCV)

Surgeries and electrochemical recordings were conducted as follows.^41^ Briefly, mice were anesthetized with isoflurane (SomnoSuite Small Animal Anesthesia System, Kent Scientific; induction 2.5%, maintenance 0.8–1.4% in O^2^, 0.35 l/min) and head-fixed on a stereotaxic frame (Kopf Instruments, Tujunga, CA). Puralube vet ointment was applied on the eye to prevent cornea from drying out. Stereotactic drill (0.8 mm) was used to preform craniotomy (unilateral, right) to target the midbrain and dorsal striatum with the following coordinates^69^ (values are in mm from Bregma); midbrain: anteroposterior = −2.9, mediolateral=+1.0, dorsoventral=+4; Dorsal Striatum: anteroposterior = +1.2, mediolateral = +1.3, dorsoventral = +3.1. An Ag/AgCl reference electrode via a saline bridge was placed under the skin. For electrical stimulations, a 22G bipolar stimulating electrode (P1 Technologies, VA, USA) was lowered to target ventral midbrain (between 4–4.5mm). The exact depth was adjusted for maximal dopamine release. For recording the evoked dopamine release, a custom-built carbon fiber electrode (5 μm diameter, cut to ~150 μm length, Hexcel Corporation, CT, USA) was lowered to reach dorsal striatum. Dil-coated carbon-fiber electrodes were used to identify the electrode position in the dorsal striatum and the electrode track in the brain tissue identified the position of the stimulation electrode (Figure S8C). The evoked dopamine release was measured using constant current (400μA), delivered using an Iso-Flex stimulus isolator triggered by a Master-9 pulse generator (AMPI, Jerusalem, Israel). A single burst stimulation consisted of 30 pulses at 50Hz (0.6s). Electrodes were calibrated using known concentration of dopamine in ACSF. Custom-written procedure in IGOR Pro was used for the data acquisition and analysis.

#### Computational model of dopamine reuptake and release

A computational model comprised of a system of ordinary differential equations (ODEs) was adapted from our previous study.^42^ The model simulates the release of dopamine *in vivo* from synaptic vesicles into the dorsal striatum and the measurement of dopamine at the carbon-fiber electrode:

(Equation 1) $$\frac{d[DA{]}_{S}}{dt}=DA_{P}IfSL-\frac{V_{m}[DA{]}_{S}}{[DA{]}_{S}+K_{m}}$$

(Equation 2) $$S(t)=\sum_{i}\theta \left(t-t_{i}\right)\theta \left(t_{i}+\frac{NP}{f}-t\right)$$

(Equation 3) $$\frac{d[DA{]}_{E}}{dt}=k_{S}[DA{]}_{S}-k_{E}[DA{]}_{E}+k_{\Gamma }\Gamma_{DA}$$

(Equation 4) $$\frac{d\Gamma_{DA}}{dt}=k_{1}^{ads\;}[DA{]}_{E}-k_{2}^{ads\;}[DA{]}_{E}\Gamma_{DA}-k_{3}^{ads\;}\Gamma_{DA}$$

The concentration of dopamine in the striatum $[DA{]}_{S}$ is computed as the difference between dopamine released into the extracellular space and dopamine removed through reuptake by DAT. *DA_P_* is the amount of dopamine release per electrical stimulus pulse, while I and f are the stimulus current (in mA) and stimulus frequency (in Hz) of the experimental protocol. The electrical pulse trains are modeled using the stimulation pattem S, where θ is the Heaviside theta function, $t_{i}$ is the start time of the stimulus, and NP is the number of electrical pulses. DAT uptake is modeled using first-order Michaelis-Menten kinetics,^72,73^ with $V_{m}$ and $K_{m}$ as the maximal velocity and affinity constant of dopamine. A loss factor L<1 is used to factor in diffusion through the dead space, a region of damaged tissue that is formed when the electrode is inserted into the tissue.^74^

The concentration of dopamine measured at the electrode $[DA{]}_{E}$ is computed as the difference between dopamine that arrives from the striatum and is oxidized and the oxidized dopamine that is reduced and “bounces off” the carbon-fiber electrode, which acts as a reflective surface.^75^ The slow temporal response of FSCV electrodes is written using first-order reactions,^75^ with $k_{S}$ and $k_{E}$ as the rate transfers of dopamine moving from the striatum and away from the electrode, respectively. Additionally, to model the electrochemical adsorption that occurs with carbon-fiber electrodes,^76^ the concentration of dopamine adsorption $\Gamma_{DA}$ is computed as the difference between the dopamine that adsorbs and desorbs to the electrode, and it is included in the calculation of $[DA{]}_{E}$ with $k_{\Gamma }=1$ as the rate transfer of adsorption. $k_{1}^{ads\;}$ is the adsorption kinetic rate constant, while $k_{2}^{ads\;}$ and $k_{3}^{ads\;}$ are the desorption kinetic rate constants.

#### *Ex vivo* dichloropane-DAT imaging and quantitation

Dichloropane, a DAT ligand, was conjugated with rhodamine red-X as described by Fiala et al.,^44^ to obtain dichloropane–rhodamine red-X probe (dichloropane probe). Mice (n=5/genotype) were sacrificed by cervical dislocation under isoflurane anesthesia and the brains were quickly dissected. Coronal slices (300 μm) of striatum were cut (VT1200S, Leica) in ice-cold carbogenated solution containing: 100 mM choline chloride, 25 mM NaHCO_3_, 1.25 mM NaH_2_PO_4_, 2.5 mM KCl, 7 mM MgCl_2_, 0.5 mM CaCl_2_, 15 mM glucose, 11.6 mM sodium ascorbate, and 3.1 mM sodium pyruvate. Striatal slices were incubated at 37°C (30 mins) in carbogenated ACSF containing: 127 mM NaCl, 25 mM NaHCO_3_, 1.25 mM NaH_2_PO_4_, 2.5 mM KCl, 1 mM MgCl_2_, 2 mM CaCl_2_ and 15 mM glucose. Slices were then warmed to room temperature in carbogenated ACSF and incubated in dichloropane probe (100 nM) for 45 mins at 37°C. Following this, slices were washed once in carbogenated ACSF and the dorsal striata maintained in ACSF were imaged under 25X water immersion objective at 561 nm excitation using a confocal microscope (LSM 900, Zeiss). Images were analyzed blind to the genotype for the presence of large DAT^+^ structures and the number of DAT^+^ puncta using FIJI software.

#### Electron microscopy

3-month-old mice brains (n=2–3/genotype) were fixed by intracardial perfusion using 2% PFA and 2% glutaraldehyde prepared in 0.1M PB, followed by overnight immersion in 0.1 M cacodylate buffer with 2.5 % of glutaraldehyde and 2 % PFA. For DAT-immunogold labeling (15 nm gold particles, DAT antibody-Synaptic Systems 284005), we used 3% PFA prepared in 1X PBS for intracardial perfusion, and 2% PFA and 0.15% glutaraldehyde prepared in 1X PBS for immersion fixation. Dorsal striatum was dissected, further processed at the Yale Center for Cellular and Molecular Imaging, Electron Microscopy Facility. EM imaging was performed using FEI Tecnai G2 Spirit BioTwin Electron Microscope. Images were analyzed blinded to the genotype using FIJI software for synaptic autophagosomes, both in symmetric and asymmetric synapses. Similarly, synaptic CCVs and SVs per synapse were also counted, along with examining the images for axonal whirls and early autophagic vacuoles. For DAT-immunogold labeling, when 5 or more immunogold^+^ DAT are located close (0–30 nm) to each other, we considered it as ‘cluster’.

For EM of purified CCVs and clathrin cages, buffer containing CCVs was pipetted onto a parafilm containing glutaraldehyde and uranyl acetate to make a 18% glutaraldehyde and 73% uranyl acetate solution in 1X PBS. EM grids were floated on top of pipetted droplets and then dried for imaging, using Philips 301 Electron Microscope. Diameter of the CCVs and empty clathrin cages, as well as their numbers were measured using iTEM software (ResAlta Research Technologies, USA).

### QUANTIFICATION AND STATISTICAL ANALYSIS

For behavioral studies, two-way ANOVA followed by Sidak’s multiple comparison test was used. For all other experiments, Student’s t-test with Welch’s correction was used. Number of mice used for each experiment are described as “n” in Results and figure legends. Values are expressed as mean ± standard error of the mean (SEM) and p value of 0.05 or less was considered statistically significant. Student’s t-test was also used to check if there are sex-based differences in the experimental results within auxilin KO mice, which was not significant. We used GraphPad Prism (9.2.0) software to perform statistical analyses.

## Supplementary Material

1

2

3

## Figures and Tables

**KEY RESOURCES TABLE:** 

REAGENT or RESOURCE	SOURCE	IDENTIFIER

Antibodies		

Rabbit Anti-TH	Millipore	Cat# AB152; RRID: AB_390204
Mouse Anti-TH	Synaptic Systems	Cat#. 213211; RRID. AB_2636901
Rabbit Anti-Iba1	Wako Chemicals	Cat# 019-19741) RRID. AB_839504
Guinea Pig Anti-GFAP	Synaptic Systems	Cat# 173004; RRID: AB_10641162
Guinea Pig Anti-DAT	Synaptic Systems	Cat# 284005; RRID: AB_2620019
Rabbit Anti-α-synuclein (phospho S129)	Abcam	Cat# ab51253; RRID: AB_869973
Mouse Anti-Clathrin light chain	Synaptic Systems	Cat# 113011; RRID: AB_887706
Rabbit Anti-Synaptogyrin 3	Synaptic Systems	Cat# 103303; RRID: AB_2619753
Rabbit Anti-VGAT	Synaptic Systems	Cat# 131002; RRID: AB_887871
Guinea Pig Anti-VGLUT2	Synaptic Systems	Cat# 135418; RRID: AB_2864786
Rat Anti-Hsc70	Enzo	Cat# ADI-SPA-815-D; RRID: AB_2039279
Rabbit Anti-Endophilin-A1	Synaptic Systems	Cat# 159002; RRID: AB_887757
Guinea Pig Anti-NeuN	Millipore	Cat# ABN90; RRID: AB_11205592
Rabbit Anti-LC3B	Abcam	Cat# 192890; RRID: AB_2827794
Mouse Anti-p62/SQSTM1	Novus	Cat# H00008878-M01; RRID: AB_548364
Rabbit Anti-auxilin	De Camilli lab, Yale University	N/A
Mouse Anti-β-actin	Genetex	Cat# GTX629630; RRID: AB_2728646
Mouse Anti-synaptobrevin-2	Synaptic Systems	Cat# 104211; RRID: AB_887811
Rabbit Anti-LC3B	Sigma-Aldrich	Cat# L7543; RRID: AB_796155
Biotin-conjugated secondary antibody	Vector Laboratories	Cat# PK-6101

Chemicals, peptides, and recombinant proteins		

L-DOPA	Sigma-Aldrich	Cat# D9628
Dichloropane-rhodamine red-X probe	Sames Lab, Columbia University, Fiala et al., 2020	https://doi.org/10.1021/jacs.0c00861
3,3’-diaminobenzidine	Fluka	Cat# 32750
Antifade mounting medium with DAPI	Vectashield	Cat# H-1000
Antifade mounting medium without DAPI	Vectashield	Cat# H-1200

Deposited data		

Mass spectrometry/proteomics	This paper	http://www.ebi.ac.uk/pride/archive/projects/PXD040141; Accession number: PXD040141

Experimental models: Organisms/strains		

Auxilin knock-out mice	Greene Lab, NHLB, NIH, Yim et al., 2010	https://doi.org/10.1073/pnas.100073810
C57BL/6J	The Jackson Laboratory, www.jax.org/strain/000664	Strain# 000664; RRID: IMSR_JAX:000664

Software and algorithms		

Noldus Ethovision XT	Noldus	Version 14; https://www.noldus.com/ethovision-xt; RRID: SCR_000441
StereoInvestigator	Micro-brightfield Inc.	Version 8.1; http://www.mbfbioscience.com/stereo-investigator; RRID: SCR_002526
FIJI	National Institute of Health (NIH)	Version 2.10.0; https://imagej.net/software/fiji/; RRID: SCR_002285
GraphPad Prism	Dotmatics	Version 9.2.0; www.graphpad.com/scientific-software/prism/
Qlucore Omics Explorer	Qlucore	Version 3.5; https://qlucore.com/omics-explorer
Ingenuity Pathway Analysis (IPA)	Qiagen	https://www.qiagen.com/us/products/discovery-and-translational-research/next-generation-sequencing/informatics-and-data/interpretation-content-databases/ingenuity-pathway-analysis; RRID: SCR_008653
iTEM electron microscopy image analysis	ResAlta Research Technologies	www.ResAltaTech.com
IGOR Pro	IGOR Pro	Version 6.02; http://www.wavemetrics.com/products/igorpro/Backspace; RRID: SCR_000325

## Highlights

- Auxilin KO mice recapitulate key features of Parkinson’s disease and respond to L-DOPA
- SV sorting defects lead to cytoplasmic dopamine accumulation in dorsal striatum
- DAT is trapped in the axonal deformities, leading to dopamine reuptake deficits
- Increased dopaminergic synaptic autophagy may also contribute to disease pathology
