# Supplementary material for: Dopamine transporter and synaptic vesicle sorting defects underlie auxilin-associated Parkinson’s disease
Source: Cell Rep. Author manuscript; Available in PMC 2023 Apr 25. (PMC10127800; doi:10.1016/j.celrep.2023.112231)
Supplement: 1 [file NIHMS1887292-supplement-1.pdf]

Cell Reports, Volume *42*

Supplemental information

Dopamine transporter and synaptic vesicle sorting defects underlie auxilin-associated Parkinson's disease

D.J. Vidyadhara, Mahalakshmi Somayaji, Nigel Wade, Betül Yücel, Helen Zhao, N. Shashaank, Joseph Ribaudo, Jyoti Gupta, TuKiet T. Lam, Dalibor Sames, Lois E. Greene, David L. Sulzer, and Sreeganga S. Chandra

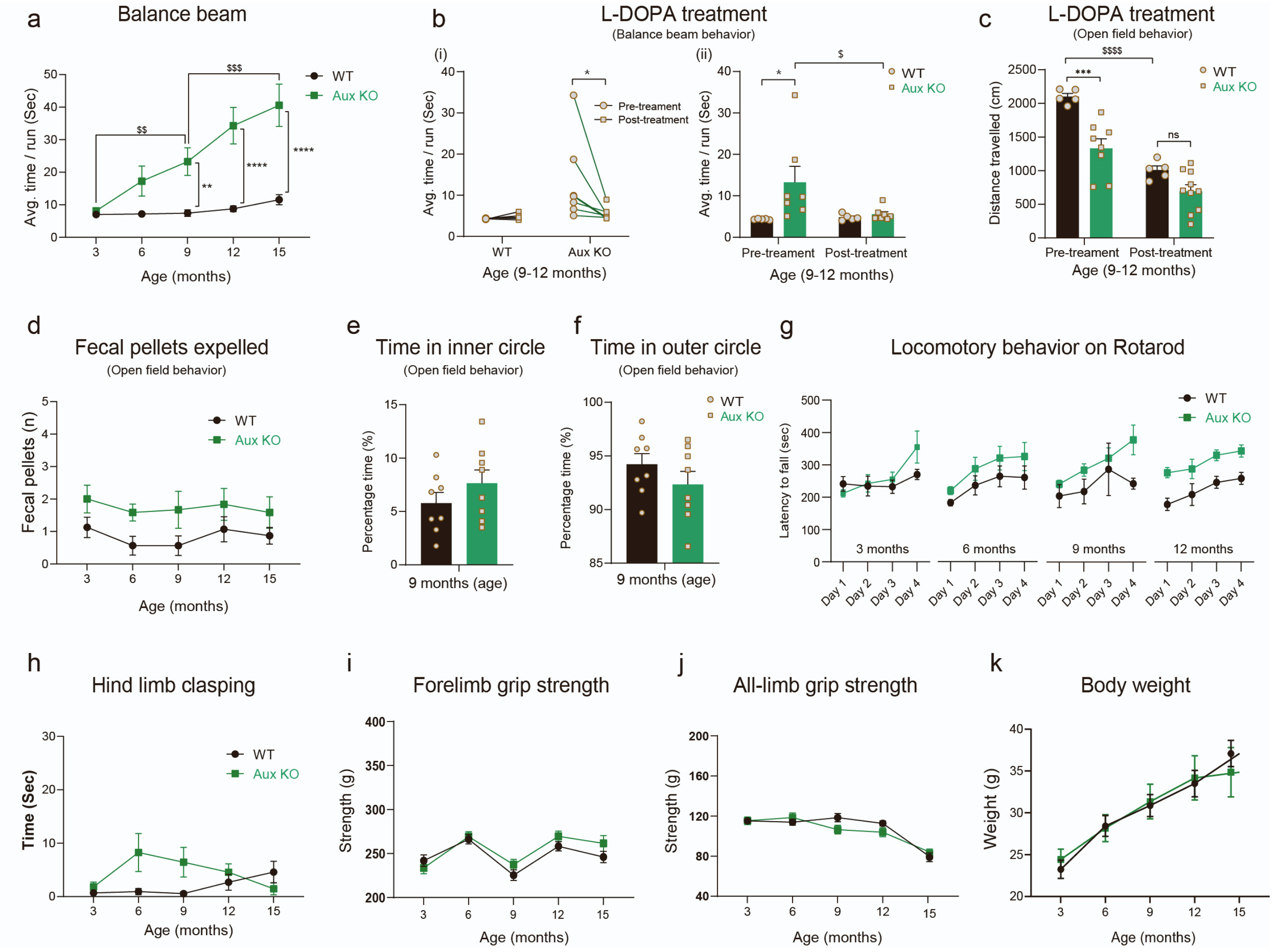

Supplemental Figure. 1: Auxilin KO mice show selective behavioral deficits and recovery to L-DOPA. Related to Figure 1. **a.** Average time taken to cross a balance beam increased with age in auxilin (Aux) KO mice. **b.** (i) Graph showing balance beam behavior (time to cross) response of individual mice after L-DOPA treatment. (ii) Time taken to cross the balance beam was restored to normal levels after L-DOPA treatment in Aux KO mice. **c.** Open field behavior pre- and post-treatment after L-DOPA. Learning and/or handling induced reduction in exploration resulted in decreased distance travelled post-treatment with L-DOPA in both WT and Aux KO mice. In the post-treatment groups, decrease in locomotory behavior in Aux KOs was not significant when compared to WT mice. **d.** Number of fecal pellets expelled during open field behavior, which did not change significantly between WT and Aux KOs across age. **e.** Percentage time spent in inner circle of the open field. This is a measure of anxiety and was not different from WT at 9 months in Aux KOs, even though motor deficits were apparent. **f.** Percentage time spent in outer circle of the open field. **g.** Motor coordination on Rotarod measured as latency to fall. Aux KOs did not show significant alteration in this behavioral test when compared to WT. **h.** No significant hind limb clasping was noted in Aux KO mice. **i.** Forelimb grip strength measured as a function of age in WT and Aux KOs. **i.** All-limb grip strength was also not affected. **j.** Body weight of WT and Aux KOs as a function of age. Statistics: For age-related behavior, two-way ANOVA followed by Sidak's multiple comparison test was used. For others, Student's t-test with Welch's correction was used. $*p<0.05$, $**p<0.01$, $****p<0.0001$, $^{\$}p<0.05$, $^{\$\$}p<0.01$, $^{\$\$\$}p<0.001$, $^{\$\$\$\$}p<0.0001$

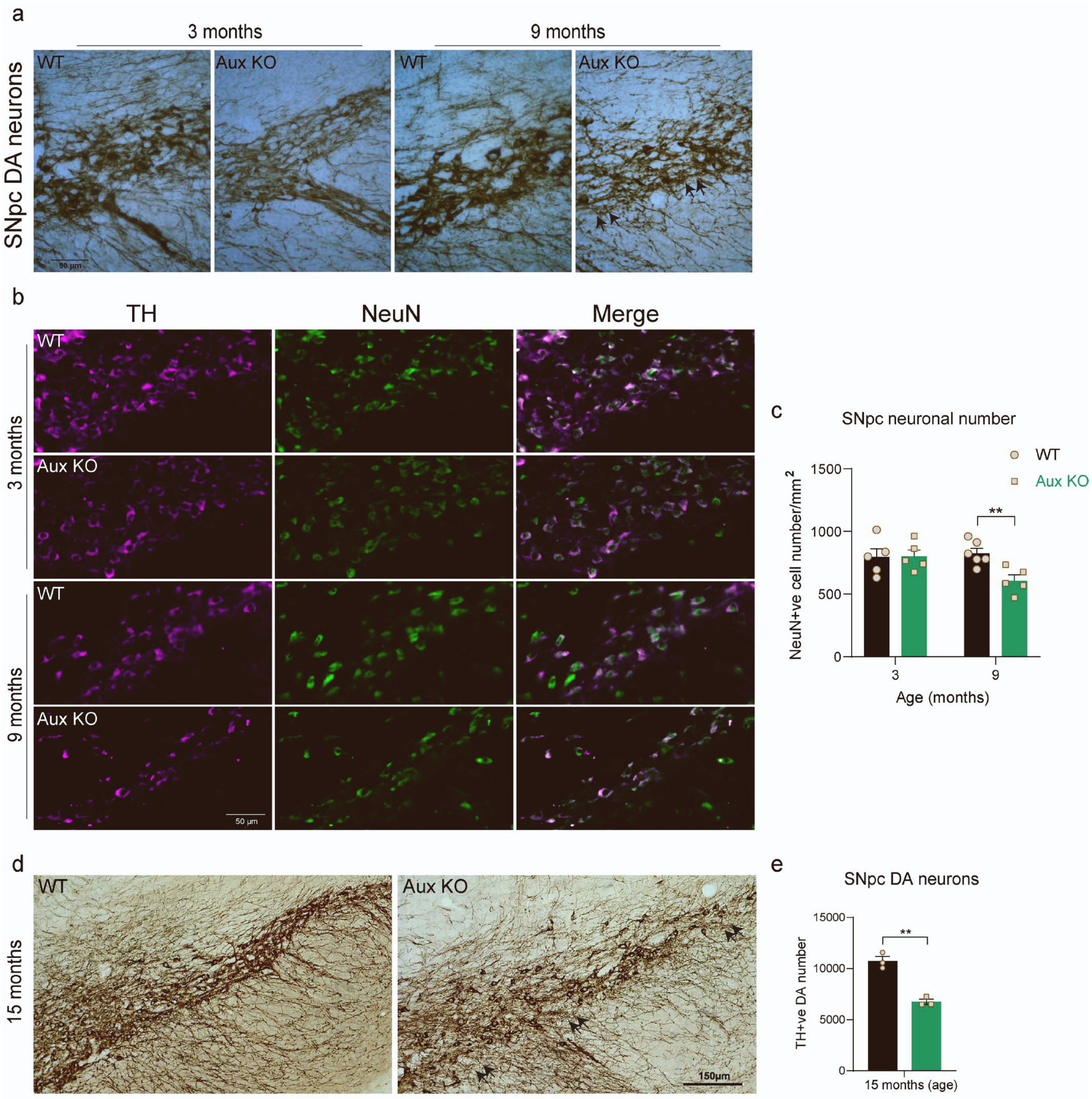

Supplemental Figure. 2: Auxilin KO mice exhibit neuronal loss in SNpc. Related to Figure 1 and 2. **a.** Representative high magnification images showing loss of TH+ve dopaminergic neurons in the SNpc of 9 month old Aux KO mice. Scale bar: 50 µm **b.** Representative images of SNpc showing TH+ve DA neurons (magenta) colocalised with neuronal marker NeuN (green) in 3 and 9 months old mice. Note a loss of both TH+ve and NeuN+ve neurons at 9 months of age in Aux KOs when compared to WT, indicating dopaminergic neuronal loss. Scale bar: 50 µm **c.** Aux KO mice showed significant loss of NeuN+ve cells at 9 months of age when compared to WT. **d.** Representative images of TH+ve DA neurons in SNpc of WT and Aux KO mice at 15 months of age. Note a loss of DA neurons in the SNpc of Aux KO mice (arrows). Scale bar: 150 µm. **i.** Stereological counting of SNpc DA neurons, which revealed a significant loss of DA neurons in Aux KO mice at 15 months of age. Statistics: Student's t-test with Welch's correction, ***p<0.01*

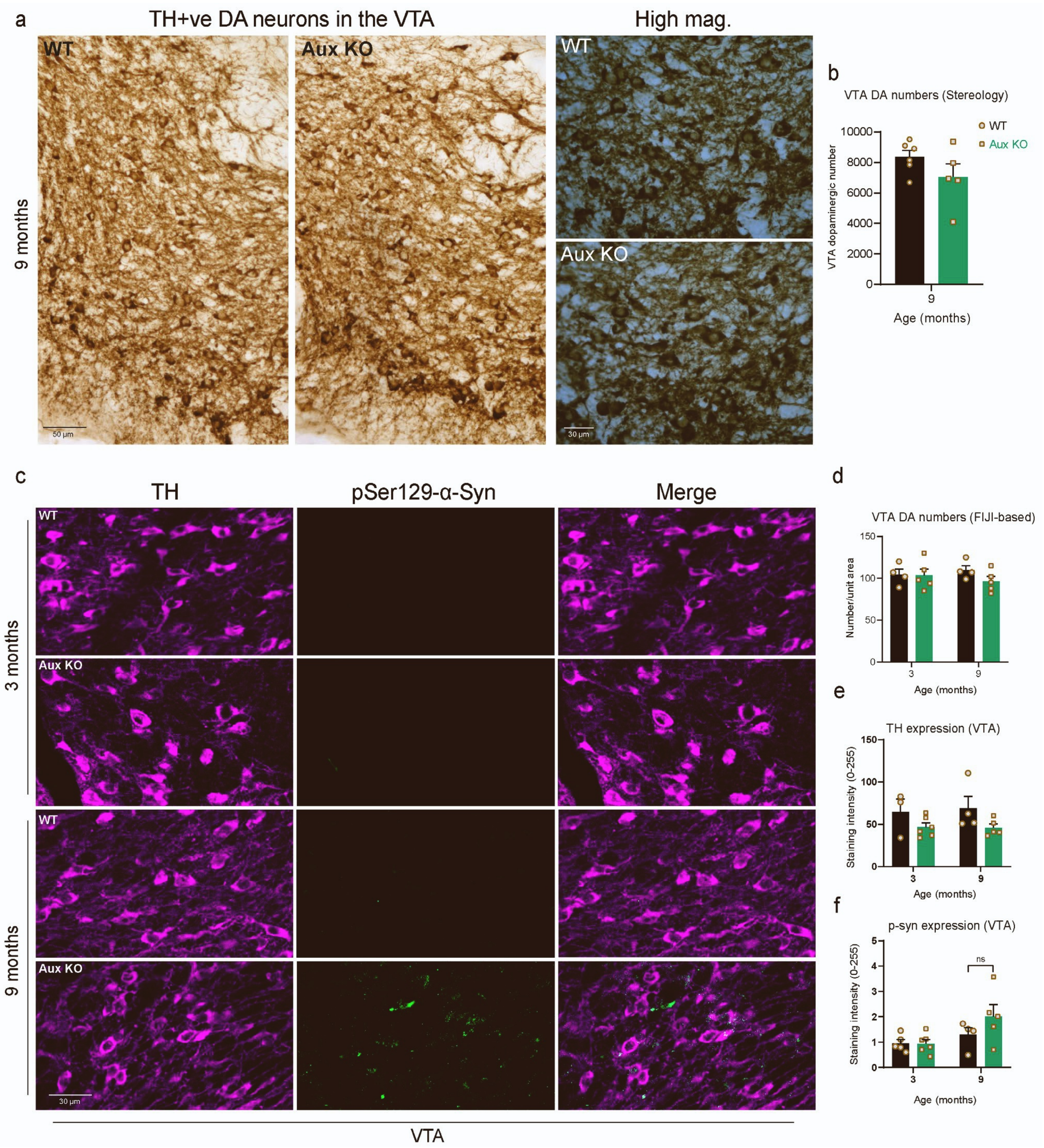

Supplemental Figure. 3: VTA is relatively preserved in auxilin KOs. Related to Figure 1 and 2. a. Representative VTA images showing TH+ve dopaminergic neurons in WT and Aux KO mice at 9 months of age. Note no difference in DA neuronal number. Scale bar: 50 µm, High mag. 30 µm b. Stereological quantitation showing no significant loss of DA neurons in the VTA of Aux KOs at 9 months of age. c. Representative images VTA immunostained for pSer129-α-synuclein (green) to denote pathology co-labelled with DA marker TH, at 3 and 9 months in WT and Aux KOs. Scale bar: 30 µm. d. TH expression in VTA, which did not change with age in Aux KOs. e. Number of TH+ve DA neurons in VTA (FIJI-based quantitation), which was not altered in Aux KO mice. f. p-Ser 129-α-synuclein expression in VTA, which showed a trend of higher expression at 9 months but did not reach significance in Aux KOs. Statistics: Student's t-test with Welch's correction. *ns = not significant*

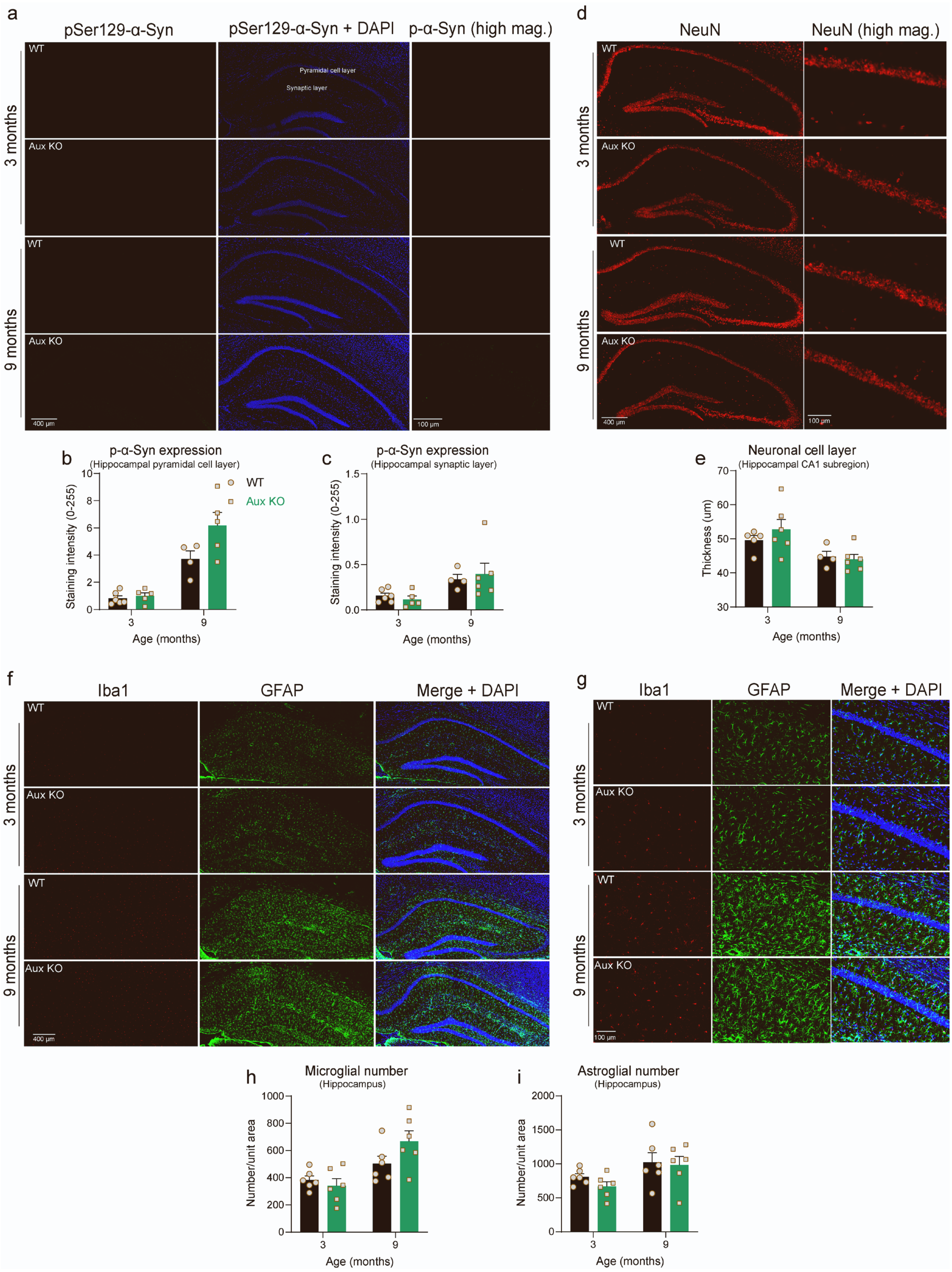

a
pSer129-α-Syn
pSer129-α-Syn + DAPI
p-α-Syn (high mag.)
3 months
9 months
WT
Aux KO
Pyramidal cell layer
Synaptic layer
400 μm
100 μm
d
NeuN
NeuN (high mag.)
b
p-α-Syn expression
(Hippocampal pyramidal cell layer)
Staining intensity (0-255)
WT
Aux KO
Age (months)
c
p-α-Syn expression
(Hippocampal synaptic layer)
e
Neuronal cell layer
(Hippocampal CA1 subregion)
Thickness (um)
f
Iba1
GFAP
Merge + DAPI
g
h
Microglial number
(Hippocampus)
Number/unit area
i
Astroglial number
(Hippocampus)

Supplemental Figure. 4: Hippocampus is preserved in auxilin KO mice. Related to Figure 2. **a.** Representative images of hippocampus immunostained for pSer129-α-synuclein (green) and DAPI at 3 and 9 months old mice. Scale bar: 400 µm. High magnification images show CA1 subregion of hippocampus. Scale bar: 100 µm **b.** A trend towards higher expression of pSer129-α-synuclein was seen in the hippocampal pyramidal cell layer of 9 month old Aux KO mice, but it was not significantly different from that of WT mice. **c.** Hippocampal synaptic layer (stratum radiatum) was spared from p-α-synuclein pathology in Aux KO mice. **d.** Representative images of hippocampus immunostained for NeuN. High magnification images show CA1 subregion of hippocampus. Scale bar: 400 µm. There is no difference between WT and Aux KOs in the thickness of CA1 cell layer, both at 3 and 9 months of age. Scale bar: 100 µm. **e.** Hippocampal neuronal density measured as thickness of CA1 subregion in Aux KO mice was comparable to that of WT. **f.** Representative hippocampal images immunostained for microglial marker Iba1 (Red) and astroglial marker GFAP (green), co-labeled with DAPI (blue). Scale bar: 400 µm. **g.** Representative high magnification images from CA1 subregion of hippocampus immunostained for Iba1 and GFAP, along with DAPI (blue). Scale bar: 100 µm. **h.** Note no change in microglial number between WT and Aux KO mice both at 3 and 9 months of age. **i.** No astrogliosis was seen in Aux KO mice. Statistics: Student's t-test with Welch's correction.

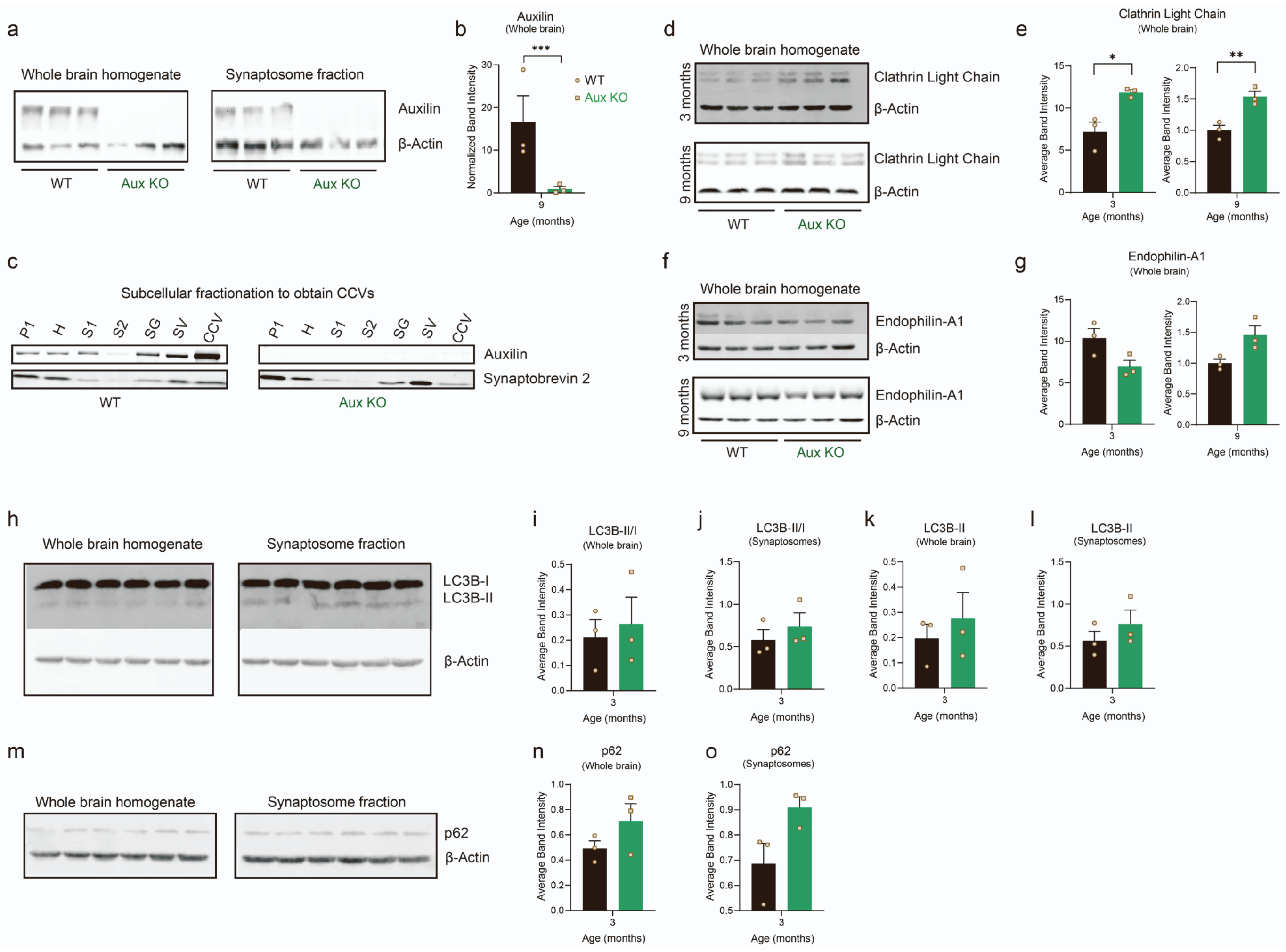

Supplemental Figure. 5: Western blots confirmed complete loss of auxilin, increased levels of clathrin, and a trend towards an increase in autophagy in the whole brain. Related to Figure 3, 5, and 7. **a**. Western blots of whole brain homogenate and synaptosome fractions showing complete loss of Aux in Aux KO mice. **b**. Quantification of auxilin expression in whole brain as measured by Western blotting. **c**. Auxilin is completely absent in all subcellular fractions obtained while preparing CCVs, whereas we see the SV protein synaptobrevin 2. P1: Pellet 1, H: Brain homogenate, S1: Supernatant 1, S2: Supernatant 2, SG: Sucrose Gradient Supernatant, SV: Synaptic vesicle fraction, CCV: CCV fraction. **d.** Western blots of Clathrin Light Chain from the whole brain samples of Aux KO and WT mice (3 and 9 months). **e.** Clathrin expression was increased in the whole brain of Aux KO mice at both 3 and 9 months of age. **f.** Western blots of Endophilin-A1 from the whole brain and synaptosome samples of Aux KO and WT mice. **g.** Endophilin-A1 showed some alterations in the expression level both at 3 and 9 months of age which was not significant. **h.** Western blots of LC3B (I and II) from the whole brain homogenate and synaptosome fractions of Aux KO and WT mice (3 months of age). **i and j.** LC3B-II/I ratio in the whole brain and synaptosomes fractions of WT and Aux KO mice. **k and l.** LC3B-II expression (autophagosome marker) showed a trend towards increased expression in the whole brain and synaptosome fractions of Aux KO mice. **n and o.** p62 showed a trend towards increased expression in the whole brain and synaptosome fraction of Aux KO mice. Part of the blot where bands were absent is digitally eliminated. Statistics: Student's t-test with Welch's correction. **p<0.05, **p<0.01, ***p<0.001*

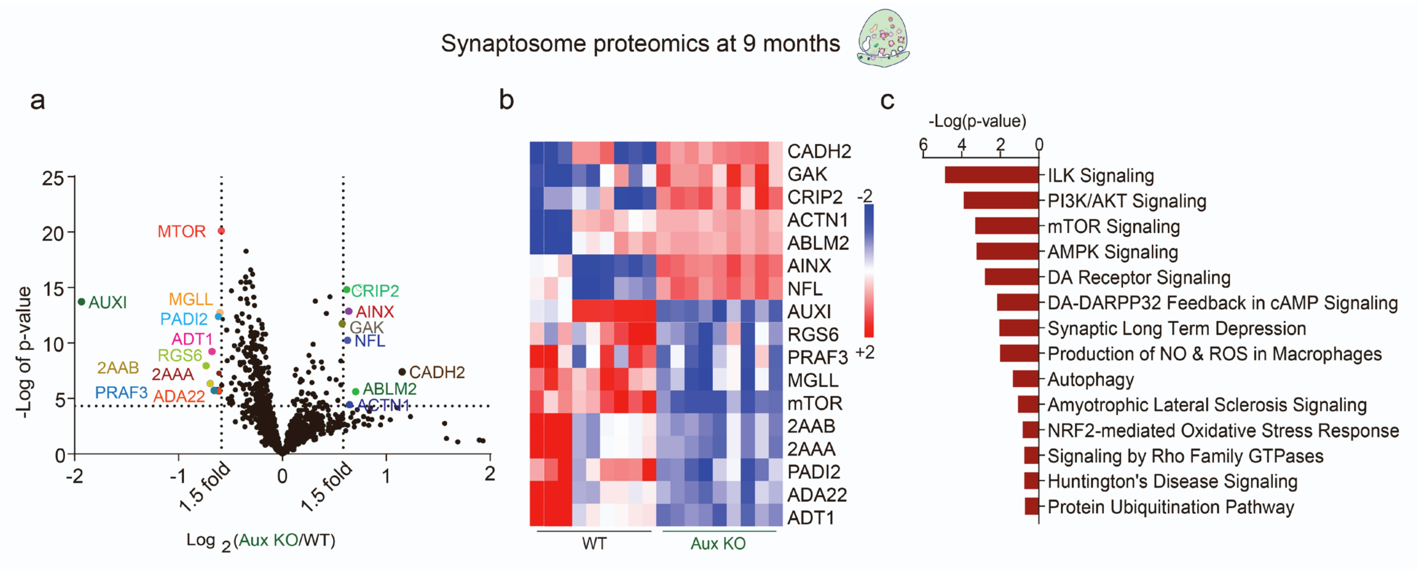

Supplemental Figure. 6: Synaptosome proteomics at 9 months revealed several PD-linked proteins and pathways were altered in auxilin KOs. Related to Figure 3. **a.** Volcano plot of synaptosome proteome of 9-month-old Aux KOs compared to WT (n=3 mice/genotype). Proteins that were altered greater than 1.5-fold (vertical dotted lines) with a p-value of 0.05 (Student's t-test) or lesser (horizontal dotted line) were considered as significantly changed. Among 17 proteins that significantly changed, 10 were decreased (left) and 7 were increased (right). **b.** Heat map of significantly changed proteins in synaptosomes of Aux KOs in comparison to WT depicted for each technical replicate (3 technical replicates/mouse). Red indicates an increase (+2) and blue indicates decreased levels (-2). **c.** Pathways that are significantly ($p<0.05$) affected in whole brain synaptosomes of Aux KOs as determined by IPA.

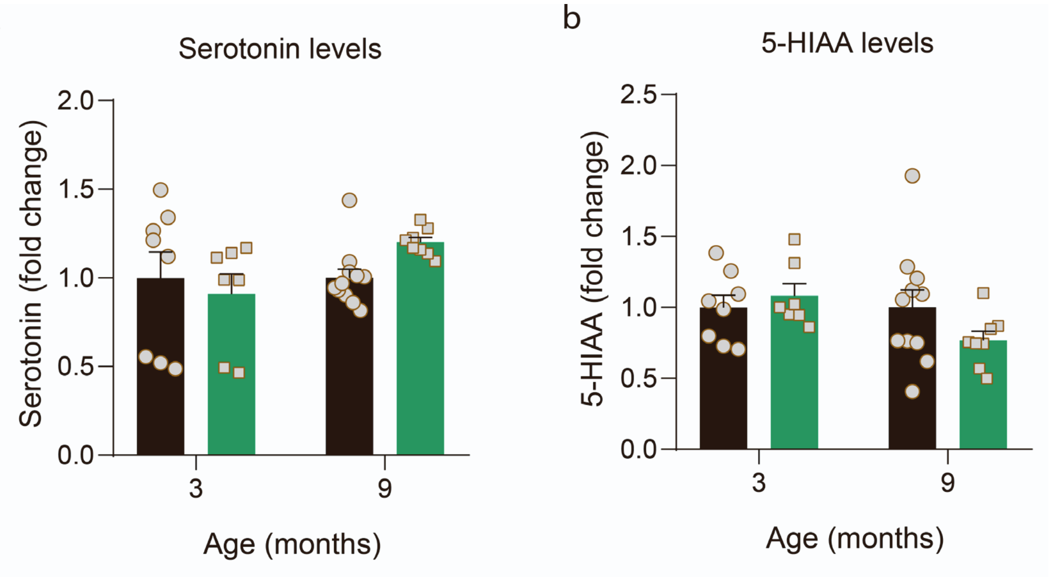

Supplemental Figure. 7: Serotonin and its metabolites were unaltered in auxilin KO brains. Related to Figure 4. **a.** Serotonin levels in the dorsal striatum of WT and Aux KOs at 3 and 9 months, as measured by HPLC, which was not altered in Aux KO mice. **b.** Levels of 5-Hydroxyindoleacetic acid (5-HIAA), a serotonin metabolite, was also unaltered. Statistics: Student's t-test with Welch's correction.

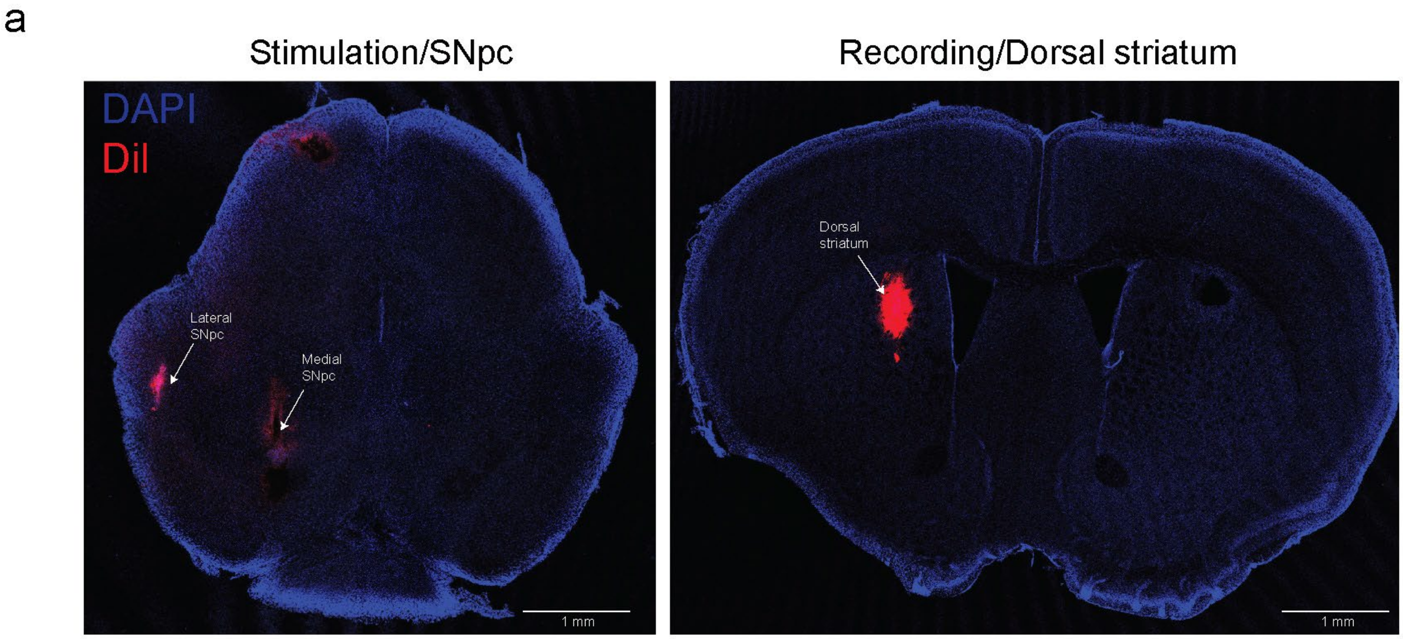

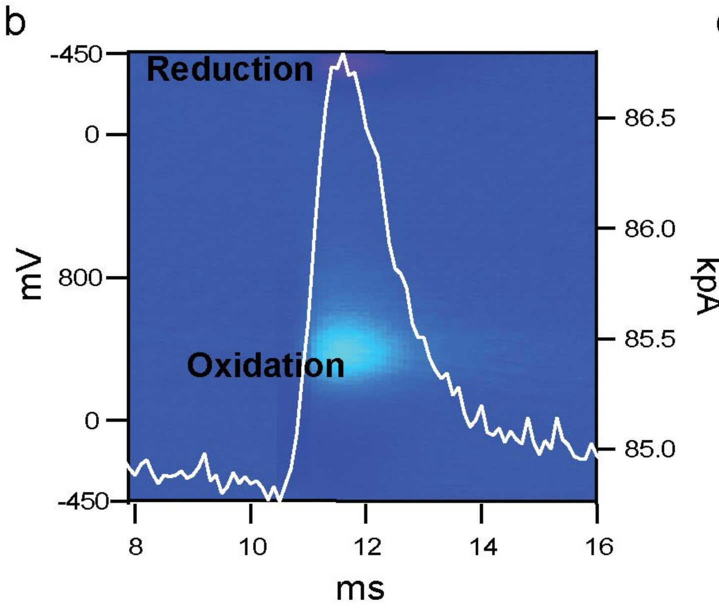

c

	WT	Auxilin KO
V_m	7.4 μM/s	2.0 μM/s
K_m	0.2 μM	0.2 μM
DA_P	0.67 μM/mA	0.31 μM/mA
I	0.4 mA	0.4 mA
f	50 Hz	50 Hz
NP	30	30
L	0.9	0.9
k_S	0.9 s^{-1}	0.9 s^{-1}
k_E	1.1 s^{-1}	0.9 s^{-1}
k_1^{ads}	0.045 s^{-1}	0.065 s^{-1}
k_2^{ads}	0.050 s^{-1}	0.070 s^{-1}
k_3^{ads}	0.080 s^{-1}	0.070 s^{-1}

Supplemental Figure. 8: FSCV recording. Related to Figure 4. **a.** Representative images of coronal mouse brain sections showing the location of the bipolar stimulating electrode in the SNpc and the FSCV recording electrode in the dorsal striatum (STR), as marked by DiI staining (DiI: red, DAPI: blue). Scale bar: 1 mm **b.** The 3-dimensional pseudocolor plot showing oxidation (cyan) and reduction (red) of dopamine. **c.** Best fit parameters of the dopamine computational model to fit FSCV recordings.

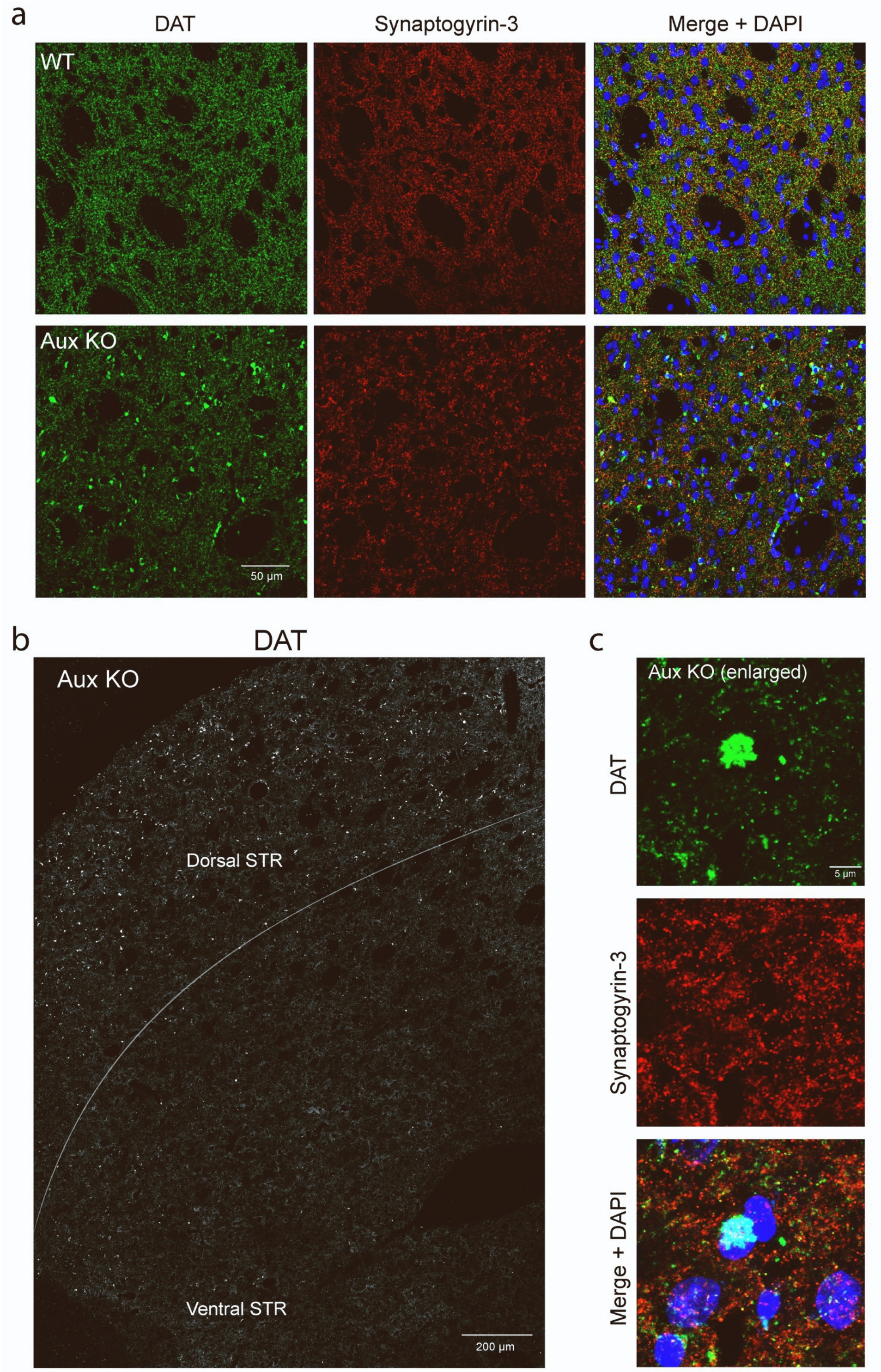

Supplemental Figure. 9: Large DAT+ structures in the dorsal striatum of auxilin KOs. Related to Figure 5. **a.** Representative images of the dorsal striatum of WT and Aux KO mice (3 months of age) immunostained for DAT (green) and synaptogyrin-3 (red). Note many large DAT+ve structures in the dorsal striatum of Aux KO, which are not seen in WT. Scale bar: 50 µm **b.** Representative grayscale image of striatum of Aux KOs immunostained for DAT, showing large DAT+ structures are enriched in the dorsolateral striatum (STR), but not in the ventral STR. Scale bar: 200 µm. **c**. Enlarged image of DAT+ve structures (green) in the dorsal striatum, co-immunostained with synapogyrin-3 (red). Scale bar: 5 µm.

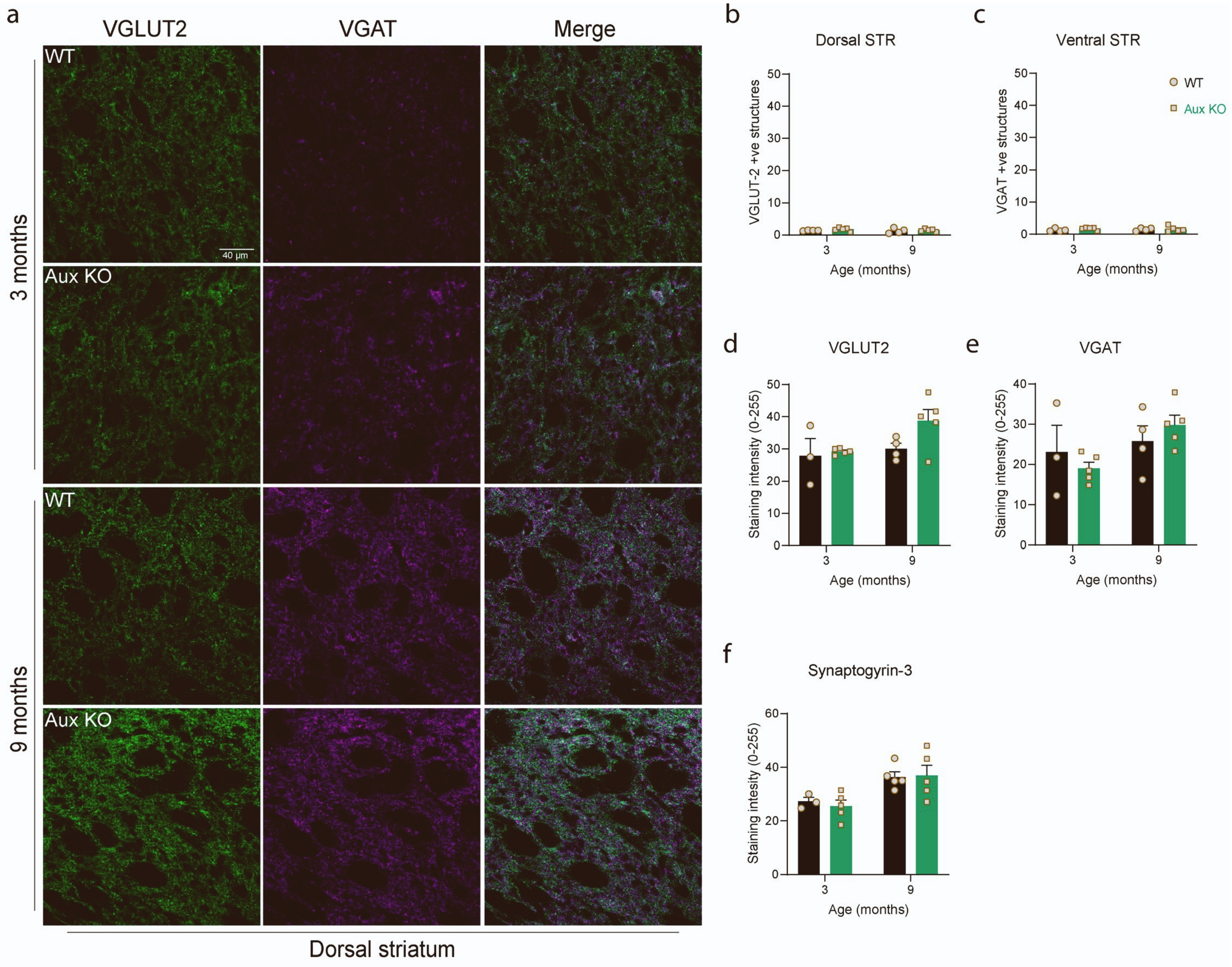

Supplemental Figure. 10: Axonal deformities were not seen in glutamatergic and GABAergic termini. Related to Figure 5. **a.** Representative images showing dorsal striatum immunostained for glutamatergic marker VGLUT2 and GABAergic marker VGAT, at 3 and 9 months of age, in WT and Aux KO mice. Scale bar: 40 µm. **b.** Quantitation for VGLUT2+ve large structures/whirls in dorsal striatum of WT and Aux KO mice. We did not observe any differences between the two genotypes. **c.** Quantitation for VGAT+ve structures in dorsal striatum of Aux KO mice which revealed no alterations. **d.** Expression of VGLUT2 in the dorsal striatum of WT and Aux KO mice at 3 and 9 months. **e.** Expression of VGAT in the dorsal striatum of WT and Aux KO mice at 3 and 9 months, which did not alter in Aux KOs. **f.** Synaptogyrin-3 expression in the dorsal striatum of WT and Aux KO mice at 3 and 9 months, which was unaltered in Aux KOs (See Figure. 4 for representative images). Statistics: Student's t-test with Welch's correction.

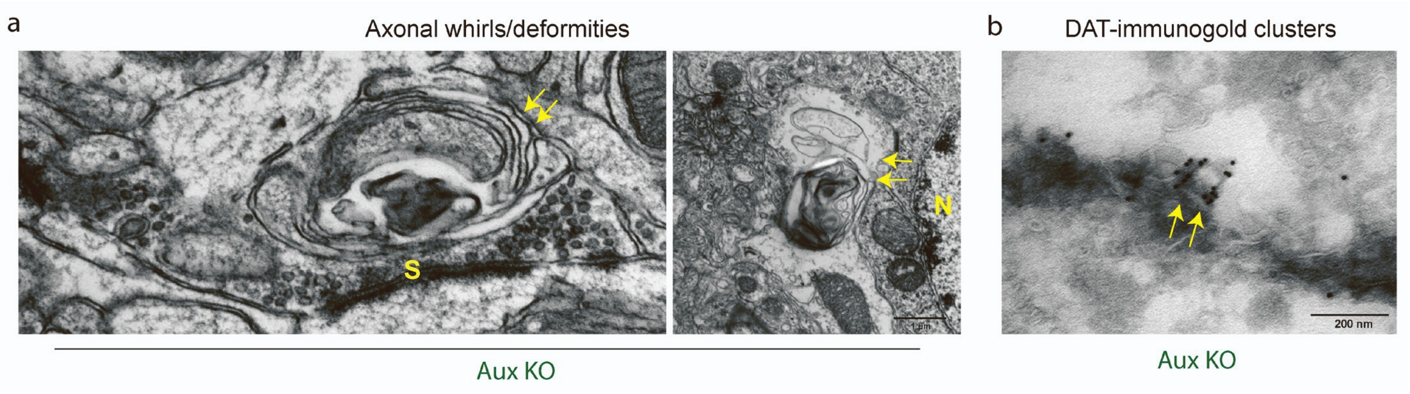

Supplemental Figure. 11: Auxilin KO mice show large axonal whirls/deformities in the dorsal striatum. Related to Figure 5. **a.** Ultrastructure of axonal whirls/deformities in the dorsal striatum of Aux KO mice (arrows). These structures were present both close to synaptic termini (S) and soma (as identified by nucleus, N)**.** Scale bar: 1 µm. **b.** DAT-immunogold clusters in the dorsal striatum of Aux KO mice (arrows). Scale bar: 200 nm.

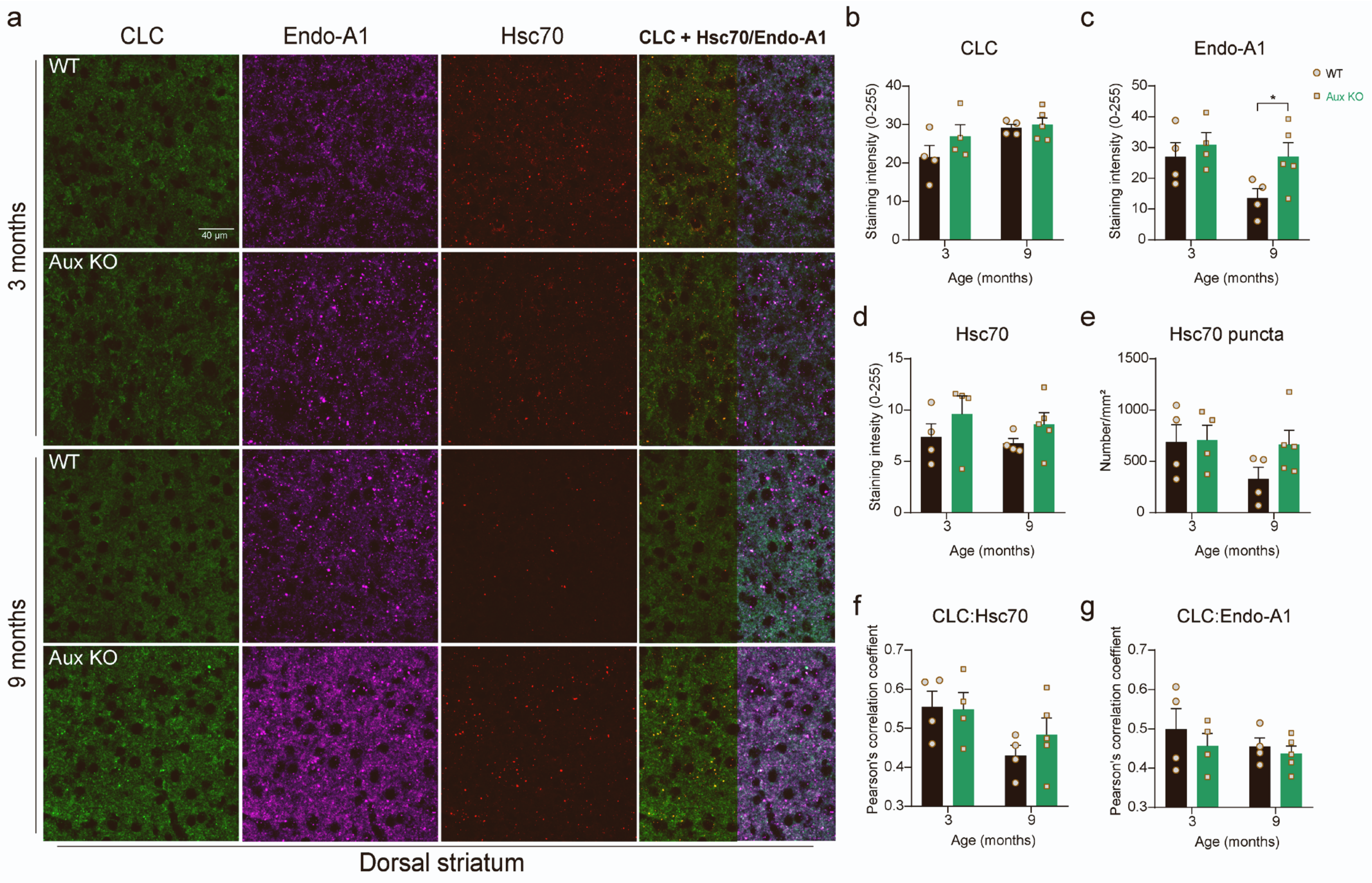

Supplemental Figure. 12: Auxilin KO mice show minimum alteration in endocytic proteins in the dorsal striatum. Related to Figure 5 and 6. **a.** Representative images of dorsal striatum immunostained for clathrin light chain (CLC), endophilin-A1 (Endo-A1) and Hsc70, in WT and Aux KO mice at 3 and 9 months of age. Scale bar: 40 µm. **b.** CLC expression the dorsal striatum, was not altered in Aux KOs. **c.** Endo-A1 expression in the dorsal striatum, was not changed at 3 months but was increased at 9 months in Aux KOs. **d.** Hsc70 expression in the dorsal striatum, which was also not altered**. e.** Number of Hsc70+ve puncta in the dorsal striatum is unaltered. **f.** Colocalization of Hsc70 with CLC in WT and Aux KOs. **g.** Endo-A1 colocalization with CLC. Statistics: Student's t-test with Welch's correction. **p<0.05*

Supplemental Table 1: Proteins that are significantly changed in the proteomic analysis of brains of auxilin KO mice, in comparison to WT (age: 3 months) (Related to Figure 3).

No.	Accession ID	Gene Name	Protein Name	fold change
1	NNTM	Nnt	NAD(P) transhydrogenase, mitochondrial	-29.7108
2	AUXI	Dnajc6	Putative tyrosine protein phosphatase auxilin	-4.43618
3	HEBP1	Hebp1	Heme-binding protein 1	-3.09322
4	WDFY1	Wdfy1	WD repeat and FYVE domain-containing protein 1	-2.63747
5	TBCD	Tbcd	Tubulin-specific chaperone D	-2.05534
6	AL7A1	Aldh7a1	Alpha-aminoadipic semialdehyde dehydrogenase	-1.77566
7	ACAP2	Acap2	Arf-GAP with coiled-coil, ANK repeat and PH domain-containing protein 2	-1.55571
8	RAB3B	Rab3b	Ras-related protein Rab-3B	-1.5479
9	NLGN4	Nlgn4l	Neuroligin 4-like	1.531757
10	NMRL1	Nmral1	NmrA-like family domain-containing protein 1	1.551981
11	ODBA	Bckdha	2-oxoisovalerate dehydrogenase subunit alpha	1.56953
12	PACS2	Pacs2	Phosphofurin acidic cluster sorting protein 2	1.575139
13	PURG	Purg	Purine-rich element-binding protein gamma	1.591686
14	CRYAB	Cryab	Alpha-crystallin B chain	1.592596
15	SPIR1	Spire1	Protein spire homolog 1	1.804854
16	SYPM	Pars2	Probable proline--tRNA ligase	1.916042
17	PRIO	Prnp	Major prion protein	2.101816
18	GAK	Gak	Cyclin-G-associated kinase	2.335293
19	ZNRD2	Znrd2	Protein ZNRD2	2.57939
20	MTND	Adi1	1,2-dihydroxy-3-keto-5-methylthiopentene dioxygenase	2.843007
21	IGG2B	Igh-3	Ig gamma-2B chain C region	3.601283
22	PURA2	Adss2	Adenylosuccinate synthetase isozyme 2	4.116065

Supplemental Table 2: Proteins that are significantly changed in the proteomic analysis of synaptosomes prepared from the brains of auxilin KO mice, in comparison to WT (age: 3 months) (Related to Figure 3).

No.	Accession	Gene Name	Protein Name	fold change
1	AUXI	Dnajc6	Putative tyrosine-protein phosphatase auxilin	-5.93229
2	NNTM	Nnt	NAD(P) transhydrogenase	-3.37721
3	HEBP1	Hebp1	Heme-binding protein 1	-3.2288
4	GBRA2	Gabra2	Gamma-aminobutyric acid receptor subunit alpha-2	-2.5017
5	WDFY1	Wdfy1	WD repeat and FYVE domain-containing protein 1	-2.19771
6	APC	Apc	Adenomatous polyposis coli protein	-1.70123
7	KCNJ4	Kcnj4	Inward rectifier potassium channel 4	-1.60661
8	COMT	Comt	Catechol O-methyltransferase	-1.59569
9	AL7A1	Aldh7a1	Alpha-aminoadipic semialdehyde dehydrogenase	-1.56755
10	IVD	Ivd	Isovaleryl-CoA dehydrogenaseIvd, mitochondrial	-1.53054
11	HTRA1	Htra1	Serine protease HTRA1	1.518369
12	CBPM	Cpm	Carboxypeptidase M	1.520221
13	NFH	Nefh	Neurofilament heavy polypeptide	1.533002
14	IMDH2	Impdh2	Inosine-5'-monophosphate dehydrogenase 2	1.573405
15	THS7A	Thsd7a	Thrombospondin type-1 domain-containing protein 7A	1.573839
16	AINX	Ina	Alpha-internexin	1.57441
17	NFL	Nefl	Neurofilament light polypeptide	1.59067
18	AP1S1	Ap1s1	AP-1 complex subunit sigma-1A	1.618179
19	SCN1A	Scn1a	Sodium channel protein type 1	1.636414
20	CH082	*N/A*	UPF0598 protein C8orf82 homolog	1.726623
21	PP2AB	Ppp2cb	Serine/threonine-protein phosphatase 2A catalytic subunit beta isoform	1.789953
22	PURA2	Adss2	Adenylosuccinate synthetase isozyme 2	2.577984
23	S61A2	Sec61a2	Protein transport protein Sec61 subunit alpha isoform 2	2.644442
24	MTND	Adi1	1,2-dihydroxy-3-keto-5-methylthiopentene dioxygenase	4.038564

Supplemental Table 3: Proteins that are significantly changed in the proteomic analysis of CCVs prepared from the brains of auxilin KO mice, in comparison to WT (age: 3 months) (Related to Figure 6).

No.	Accession	Gene Name	Protein Name	fold change
1	AUXI	Dnajc6	Putative tyrosine-protein phosphatase auxilin	-44.906
2	IGHM	IGHM	Ig mu chain C region	-3.12027
3	CD34	Cd34	Hematopoietic progenitor cell antigen CD34	-2.50216
4	NECP1	Necap1	Adaptin ear-binding coat-associated protein 1	-2.35292
5	SNG3	Syngr3	Synaptogyrin-3	-2.10263
6	HS71A	Hspa1a	Heat shock 70 kDa protein 1A	-2.02752
7	SV2B	Sv2b	Synaptic vesicle glycoprotein 2B	-1.9614
8	SPRL1	Sparcl1	SPARC-like protein 1	-1.94395
9	GRM2	Grm2	Metabotropic glutamate receptor 2	-1.9365
10	IGKC	Igkc	Ig kappa chain C region	-1.92748
11	TM163	Tmem163	Transmembrane protein 163	-1.92239
12	TCPB	Cct2	T-complex protein 1 subunit beta	-1.8972
13	SNG1	Syngr1	Synaptogyrin-1	-1.8946
14	NECP2	Necap2	Adaptin ear-binding coat-associated protein 2	-1.89401
15	ROAA	Hnrnpab	Heterogeneous nuclear ribonucleoprotein A/B	-1.86664
16	RAB3B	Rab3b	Ras-related protein Rab-3B	-1.85144
17	VGLU1	Slc17a7	Vesicular glutamate transporter 1	-1.8164
18	TRFE	Tf	Serotransferrin	-1.80762
19	LAT1	Slc7a5	Large neutral amino acids transporter small subunit 1	-1.72256
20	SYPH	Syp	Synaptophysin	-1.71627
21	SV2A	Sv2a	Synaptic vesicle glycoprotein 2A	-1.71423
22	ROA0	Hnrnpa0	Heterogeneous nuclear ribonucleoprotein A0	-1.70418
23	ZNT3	Slc30a3	Zinc transporter 3	-1.70291
24	DPP10	Dpp10	Inactive dipeptidyl peptidase 10	-1.68897
25	NTRI	Ntm	Neurotrimin	-1.68091
26	SYT12	Syt12	Synaptotagmin-12	-1.67682
27	IGLO5	Iglon5	IgLON family member 5	-1.668
28	VGLU2	Slc17a6	Vesicular glutamate transporter 2	-1.66765
29	SYT1	Syt1	Synaptotagmin-1	-1.61913
30	CAD13	Cdh13	Cadherin-13	-1.58329
31	BT3L4	Btf3l4	Transcription factor BTF3 homolog 4	-1.57599
32	SCAM5	Scamp5	Secretory carrier-associated membrane protein 5	-1.57105
33	C1QB	C1qb	Complement C1q subcomponent subunit B	-1.55463
34	STON2	Ston2	Stonin-2	-1.5433
35	VAMP2	Vamp2	Vesicle-associated membrane protein 2	-1.52957
36	MFSD6	Mfsd6	Major facilitator superfamily domain-containing protein 6	-1.5266
37	RAP1A	Rap1a	Ras-related protein Rap-1A	-1.51838
38	NHRF1	Slc9a3r1	Na(+)/H(+) exchange regulatory cofactor NHE-RF1	-1.51157

39	CC50A	Tmem30a	Cell cycle control protein 50A	1.511254
40	TPC11	Trappc11	Trafficking protein particle complex subunit 11	1.588902
41	MYPR	Plp1	Myelin proteolipid protein	1.59174
42	CLVS2	Clvs2	Clavesin-2 OS=Mus musculus	1.642113
43	MOG	Mog	Myelin-oligodendrocyte glycoprotein	1.655013
44	AP3M2	Ap3m2	AP-3 complex subunit mu-2	1.657988
45	APMAP	Apmap	Adipocyte plasma membrane-associated protein	1.727314
46	TMM9B	Tmem9b	Transmembrane protein 9B	1.738029
47	H2A1B	H2ac4	Histone H2A type 1-B	1.807098
48	NDKA	Nme1	Nucleoside diphosphate kinase A	1.808866
49	PCYOX	Pcyox1	Prenylcysteine oxidase	2.039561
50	CLCN6	Clcn6	Chloride transport protein 6	2.169131
51	ERC2	Erc2	ERC protein 2	6.374011

Supplemental Table 4: Proteins that are significantly changed in the proteomic analysis of synaptosomes prepared from the brains of auxilin KO mice, in comparison to WT, at symptomatic age of 9 months (Related to Figure 3).

No.	Accession	Gene Name	Protein Name	fold change
1	AUXI	Dnajc6	Putative tyrosine-protein phosphatase auxilin	-3.82296
2	RGS6	Rgs6	Regulator of G-protein signaling 6	-1.66333
3	ADA22	Adam22	Disintegrin and metalloproteinase domain-containing protein 22	-1.61876
4	ADT1	Slc25a4	ADP/ATP translocase 1	-1.59798
5	2AAB	Ppp2r1b	Serine/threonine-protein phosphatase 2A	-1.57516
6	2AAA	Ppp2r1a	Serine/threonine-protein phosphatase 2A	-1.54334
7	PADI2	Padi2	Protein-arginine deiminase type-2	-1.53356
8	PRAF3	Arl6ip5	PRA1 family protein 3	-1.52679
9	MGLL	Mgll	Monoglyceride lipase	-1.51729
10	MTOR	Mtor	Serine/threonine-protein kinase mTOR	-1.50276
11	CRIP2	Crip2	Cysteine-rich protein 2	1.543321
12	NFL	Nefl	Neurofilament light polypeptide	1.545559
13	AINX	Ina	Alpha-internexin	1.557636
14	CADH2	Cdh2	Cadherin-2	2.225312
15	ABLM2	Ablim2	Actin-binding LIM protein 2	1.631285
16	ACTN1	Actn1	Alpha-actinin-1	1.5648206
17	GAK	GAK	Cyclin-G-associated kinase	1.4910263

Supplemental Table 5: Protein hits assigned to Ingenuity Canonical Pathways obtained by IPA analysis of whole brain WT and auxilin KO proteomics (age: 3 months) (Related to Figure 3).

**Ingenuity Canonical Pathways**	**Downregulated proteins**	**Upregulated proteins**	**Significantly dysregulated proteins (p>0.05, <1.5 fold change)**
Methylthiopropionate Biosynthesis	0/1 (0%)	1/1 (100%)	ADI1
Choline Degradation I	1/2 (50%)	0/2 (0%)	ALDH7A1
Branched-chain α-keto acid Dehydrogenase Complex	0/4 (0%)	3/4 (75%)	BCKDHA
Lysine Degradation II	1/5 (20%)	0/5 (0%)	ALDH7A1
Lysine Degradation V	1/5 (20%)	0/5 (0%)	ALDH7A1
Aldosterone Signaling in Epithelial Cells	11/158 (7%)	46/158 (29%)	CRYAB, DNAJC6
Purine Nucleotides De Novo Biosynthesis II	0/11 (0%)	9/11 (82%)	ADSS2
Ubiquinol-10 Biosynthesis (Eukaryotic)	0/17 (0%)	4/17 (24%)	BCKDHA
Histamine Degradation	1/17 (6%)	8/17 (47%)	ALDH7A1
Valine Degradation I	0/18 (0%)	14/18 (78%)	BCKDHA
Oxidative Ethanol Degradation III	2/19 (11%)	10/19 (53%)	ALDH7A1
Fatty Acid α-oxidation	1/20 (5%)	8/20 (40%)	ALDH7A1
Putrescine Degradation III	1/21 (5%)	10/21 (48%)	ALDH7A1
Ethanol Degradation IV	3/23 (13%)	11/23 (48%)	ALDH7A1
Tryptophan Degradation X (Mammalian, via Tryptamine)	1/25 (4%)	12/25 (48%)	ALDH7A1
Protein Ubiquitination Pathway	14/273 (5%)	84/273 (31%)	CRYAB, DNAJC6
Dopamine Degradation	1/30 (3%)	10/30 (33%)	ALDH7A1
Ethanol Degradation II	2/32 (6%)	14/32 (44%)	ALDH7A1
Noradrenaline and Adrenaline Degradation	1/35 (3%)	14/35 (40%)	ALDH7A1
B Cell Development	1/36 (3%)	1/36 (3%)	Ighg2b
tRNA Charging	4/39 (10%)	21/39 (54%)	PARS2
Autoimmune Thyroid Disease Signaling	0/49 (0%)	1/49 (2%)	Ighg2b
Hematopoiesis from Pluripotent Stem Cells	1/49 (2%)	1/49 (2%)	Ighg2b
Primary Immunodeficiency Signaling	2/50 (4%)	1/50 (2%)	Ighg2b

Supplemental Table 6: Protein hits assigned to Ingenuity Canonical Pathways obtained by IPA analysis of whole brain synaptosome WT and auxilin KO proteomics (age: 3 months) (Related to Figure 3).

**Ingenuity Canonical Pathways**	**Downregulated proteins**	**Upregulated proteins**	**Significantly dysregulated proteins (p>0.05, <1.5 fold change)**
Purine Nucleotides De Novo Biosynthesis II	2/11 (18%)	7/11 (64%)	ADSS2, IMPDH2
Dopamine Degradation	6/30 (20%)	10/30 (33%)	ALDH7A1, COMT
Noradrenaline and Adrenaline Degradation	7/35 (20%)	13/35 (37%)	ALDH7A1, COMT
Dopamine-DARPP32 Feedback in cAMP Signaling	19/163 (12%)	57/163 (35%)	KCNJ12, KCNJ4, PPP2CB
Methylthiopropionate Biosynthesis	0/1 (0%)	1/1 (100%)	ADI1
Choline Degradation I	1/2 (50%)	0/2 (0%)	ALDH7A1
L-DOPA Degradation	1/2 (50%)	0/2 (0%)	COMT
Dopamine Receptor Signaling	9/77 (12%)	29/77 (38%)	COMT, PPP2CB
CTLA4 Signaling in Cytotoxic T Lymphocytes	2/89 (2%)	26/89 (29%)	AP1S1, PPP2CB
Amyotrophic Lateral Sclerosis Signaling	17/97 (18%)	18/97 (19%)	NEFH, NEFL
Lysine Degradation II	1/5 (20%)	0/5 (0%)	ALDH7A1
Lysine Degradation V	1/5 (20%)	0/5 (0%)	ALDH7A1
Leucine Degradation I	3/9 (33%)	5/9 (56%)	IVD
Urate Biosynthesis/Inosine 5'-phosphate Degradation	2/13 (15%)	3/13 (23%)	IMPDH2
Wnt/β-catenin Signaling	10/173 (6%)	23/173 (13%)	APC, PPP2CB
Histamine Degradation	5/17 (29%)	8/17 (47%)	ALDH7A1
Xenobiotic Metabolism CAR Signaling Pathway	15/189 (8%)	38/189 (20%)	ALDH7A1, PPP2CB
Purine Nucleotides Degradation II (Aerobic)	3/18 (17%)	3/18 (17%)	IMPDH2
Oxidative Ethanol Degradation III	6/19 (32%)	10/19 (53%)	ALDH7A1
Fatty Acid α-oxidation	5/20 (25%)	8/20 (40%)	ALDH7A1
Putrescine Degradation III	5/21 (24%)	10/21 (48%)	ALDH7A1
Polyamine Regulation in Colon Cancer	2/22 (9%)	4/22 (18%)	APC
Ethanol Degradation IV	7/23 (30%)	10/23 (43%)	ALDH7A1
Tryptophan Degradation X (Mammalian, via Tryptamine)	6/25 (24%)	13/25 (52%)	ALDH7A1
Ethanol Degradation II	7/32 (22%)	13/32 (41%)	ALDH7A1
Fatty Acid β-oxidation I	4/32 (13%)	19/32 (59%)	IVD
Cell Cycle Regulation by BTG Family Proteins	2/37 (5%)	7/37 (19%)	PPP2CB
Xenobiotic Metabolism Signaling	26/287 (9%)	58/287 (20%)	ALDH7A1, PPP2CB
